# Stress-induced release of Oct-1 from the nuclear envelope is mediated by JNK phosphorylation of lamin B1

**DOI:** 10.1371/journal.pone.0177990

**Published:** 2017-05-24

**Authors:** Ivan I. Boubriak, Ashraf N. Malhas, Marek M. Drozdz, Lior Pytowski, David J. Vaux

**Affiliations:** Sir William Dunn School of Pathology, University of Oxford, Oxford, United Kingdom; North Carolina State University, UNITED STATES

## Abstract

The nuclear lamina can bind and sequester transcription factors (TFs), a function lost if the lamina is abnormal, with missing or mutant lamin proteins. We now show that TF sequestration is not all-or-nothing, but a dynamic physiological response to external signals. We show that the binding of the ubiquitous TF, Oct-1, to lamin B1 was reversed under conditions of cellular stress caused, *inter alia*, by the chemical methylating agent methylmethanesulfonate (MMS). A search for lamin B1 post-translational modifications that might mediate changes in Oct-1 binding using kinase inhibitors uncovered a role for c-Jun N-terminal kinase (JNK). Phosphoproteomic and site-directed mutagenesis analyses of lamin B1 isolated from control and MMS-treated nuclei identified T575 as a JNK site phosphorylated after stress. A new phospho-T575 specific anti-peptide antibody confirmed increased interphase cellular T575 phosphorylation after cell exposure to certain stress conditions, enabling us to conclude that lamin B1 acts as an interphase kinase target, releasing Oct-1 to execute a protective response to stress.

## Introduction

The nuclear envelope (NE) is composed of the inner and outer nuclear membranes in addition to a nucleoplasmic meshwork of proteins, the nuclear lamins, which contribute to the structural integrity of the nucleus [1]. A-type lamins are developmentally regulated and are expressed in differentiated cells, while at least one B-type lamin is expressed in all vertebrate cells. The NE is involved in various cellular processes including the organisation of chromosomes within the nucleus, DNA replication and repair, transcription, apoptosis and mitosis. There is also evidence that the NE is involved in signalling [2–6]. The importance of the NE is highlighted by the laminopathies, a group of diseases that result from mutations in genes coding for its components.

We have previously shown that lamin B1 can contribute to the control of gene expression by tethering specific chromosomes [7] and the transcription factor, Oct-1 [5]. We found that in mouse embryonic fibroblasts (MEFs) lacking full length lamin B1, Oct-1 is no longer sequestered at the NE and is therefore available to bind to its target sequences and regulate their expression [8]. Some of these targets are involved in oxidative stress responses and as a result lamin B1-deficient MEFs have elevated reactive oxygen species (ROS) levels and are susceptible to oxidative stress. Furthermore, we have shown that miRNA-31 is a target of Oct-1 through which lamin B1 and Oct-1 regulate cell cycle progression [9]. All the above experiments, however, had been performed in *Lmnb1*^Δ/Δ^ cells where the C-terminus of lamin B1 was completely absent. In the current study, we demonstrate that Oct-1 binding to lamin B1 is a controlled process in response to cellular stress. Lamin B1 Oct-1 interaction is lost under conditions of stress caused by the DNA alkylating agent methylmethanesulfonate (MMS) in a JNK-dependent manner. We report that phosphorylation of lamin B1 at T575, at least in part, by JNK in response to MMS treatment results in Oct-1 release from the NE and the downstream effects that Oct-1 has on *GADD45A* expression. This is to our knowledge the first evidence of a phosphorylation-dependent stress-signalling event involving the nuclear lamin B1.

## Materials and methods

### Cell culture and treatments

Cells, HeLa and human dermal fibroblast (HDFs) cells were grown in DMEM supplemented with 10% FCS, L-glutamine, and non-essential amino acids. BRCA1-reconstituted HCC1937 cells were generated by transfecting HCC1937 cells with full-length BRCA1-pIRES2-EGFP or full-length BRCA1 with point mutation I26A-pIRES2-EGFP (provided by X. Yu, Mayo Clinic, Rochester, MN) as previously described [10]. Drug concentrations used were as follows; Methyl methane sulfonate (MMS) 100 μg/ml, Zeocin 50 μg/ml, SP6000125 10 μM, UO126 20 μM, H_2_O_2_ 4 mM. Heat shock was performed by incubating the cells at 45°C for 10 minutes. DNA constructs used in the current study were GFP-lamin B1 [11] and GFP-Oct1 (kind gift from S. Murphy, Sir William Dunn School of Pathology). Antibodies used in the current study were anti-lamin B1 (Santa Cruz; sc-6216), anti-lamin B1 (own production 8D1) [11], anti-Oct1 (Santa Cruz; sc-232 and sc-232x), anti-Oct1 (GenTech; GTX105202) anti-GFP (Abcam; ab5449), anti-GFP (GeneTech; GT7312) anti-JNK (Cell Signalling; 9258), anti-phospho-JNK (Cell Signalling; 4668), anti-phospho-c-Jun (Cell Signalling; 9261) and anti-actin (Abcam; AC-15). Sequential extraction of nuclear proteins was performed as described previously [12]. Briefly, purified nuclei were re-suspended in nuclear isolation buffer (10 mM HEPES, pH 7.4, 2 mM MgCl_2_, 25 mM KCl, 250 mM sucrose, 1 mM DTT) and sonicated for two 5 s bursts at 10 mm amplitude. Insoluble material was re-suspended in nuclear extraction buffer (20 mM HEPES, pH 7.4, 1 M NaCl) and incubated at room temperature with agitation for 20 min. The extraction was repeated using nuclear extraction buffer with 2% Triton X-100 and 4 M urea in sequential extractions. All fractions were then analyzed by Western blotting.

### Cell transfection

DNA transfections were performed using Lipofectamine 2000 (Invitrogen), whereas siRNA transfections were performed using transfection reagent Lipofectamine RNAiMax (Invitrogen). All experiments were performed 72 h after transfection. All siRNA were purchased from Applied Biosystems

### RNA extraction and real-time PCR

All reagents and equipment were purchased from Applied Biosystems. RNA was extracted and cDNA prepared using the cells-to-cDNA II kit following manufacturer’s instructions. Real-time PCR of human *GADD45A* and Beta Actin (*ACTB*) was performed using TaqMan probes and the TaqMan Fast Universal PCR mix. All real-time PCR was performed using the Step One Plus real-time PCR system. Primer sequences are shown in S1 Table, together with statistical values for the experiments shown in the main figures as S2, S3, S4 and S5 Tables.

### Immunoprecipitation (IP)

Cells were lysed using NP-40 lysis buffer in the presence of complete protease inhibitor cocktail (Roche). Lysates were pre-cleared twice using uncoated Protein A Dynabeads and immunoprecipitated overnight at 4°C using the appropriate antibody coupled to Protein G Dynabeads (Invitrogen). Antibodies used for IP in the current study were anti-lamin B1 (Santa Cruz; C20), anti-GFP (Abcam; ab5449), and anti-GFP (GeneTech, GT7312). Following washing with binding and washing buffers from IP Dynabeads Protein G kit (Invitrogen), antigens were eluted using elution buffer with NuPage LDS sample buffer (Invitrogen) and analysed by Western blotting as indicated. In some cases, cells were crosslinked with disuccinimidyl suberate (DSS) (Pierce) or BS3 crosslinker (Abcam) followed by RIPA buffer lysis and IP as described above and results were compared to those from IP experiments without crosslinking.

### Immunostaining

HeLa cells were immunostained as described previously [13]. Briefly cells were fixed in 4% paraformaldehyde/PBS solution and after treatment with 25 mM glycine/PBS and permeabilisation in 0.5% Triton X 100/PBS, fixed cells were blocked overnight at 4°C in 0.5% fish skin gelatine/PBS solution. Rabbit anti-Oct-1 antibodies (Santa Cruz; sc-232x) and mouse anti-lamin B1 (monoclonal 8D1) antibodies were used. Secondary donkey anti-rabbit or anti-mouse IgG antibodies were conjugated to Alexa 488 (Invitrogen; A21206) or Cy-3 (Jackson Immuno Research; 715-165-150), respectively. Coverslips were mounted using Mowiol supplemented with Di-Amino-Phenyl-Indole (DAPI). Immunostaining was analysed on Zeiss LSM 880 Airyscan Confocal Microscope (Dunn School Bioimaging Unit, Oxford) in confocal or airyscan mode.

### Site-directed mutagenesis

The QuickChange II XL site-directed mutagenesis kit (Agilent) was used to change the following potential phosphorylation sites within GFP-lamin B1 into alanine and glutamic acid; S391, S393, S508 and T575. All primers required were designed using the QuickChange primer design application (Agilent). Primer sequences are shown in S7 Table.

### Fluorescence loss in photobleaching (FLIP)

FLIP was carried out as described previously [12]. Briefly, a region of interest (ROI) was photobleached at full laser power while scanning at imaging laser power elsewhere. For quantitative analysis, background intensity was subtracted and intensities of a specific ROI outside the photobleached area were measured over time and normalized using intensities of an ROI in a transfected but non-bleached cell.

### Chromatin immuoprecipitation (ChIP)

ChIP was performed using the SimpleChIP enzymatic chromatin IP kit (Cell Signaling Technology) following the manufacturer’s instructions. Detailed protocol for ChIP analysis was published elsewhere [8]. Precipitated DNA was analyzed by qRT-PCR using a Rotor-Gene 3000 (Corbett Research) and the MESA GREEN qPCR MasterMix Plus for SYBR Assay (Eurogentec). PCR mixture contained 2ul of immunoprecipitate or input samples and primers designed for the detection of the Oct-1 binding sequence within the *GADD45A* gene at final concentration of 400 nM. Relative gene expression values were determined using the 2^−ΔΔCt^ method [14]. The C_t_ values from qRT-PCR were normalized using those of the input samples and were used to calculate the fold enrichment of Oct-1 binding in control and MMS treated cells. Primers used in the ChIP study are shown in S1 Table, together with the statistical values for the four repeats of the ChIP experiment shown in S6 Table with details in parts a, b and c.

### Polyclonal phospho-specific antibody production

Rabbit anti-peptide antisera were produced by Covalab UK Ltd (Cambridge, UK) using a synthetic phospho-peptide (HQQG[Tp]PRASNRSC) as immunogen, followed by double affinity purification, with positive selection on the immunogen and negative selection on the equivalent non-phosphorylated peptide (HQQGTPRASNRSC).

### Flow cytometry

Cells were fixed overnight in methanol at -20°C, permeabilised using 0.25% Triton X-100/ PBS and labelled with rabbit anti-phospho-lamin B1 followed by donkey anti-rabbit Alexa 488. Cells were then resuspended in propidium iodide (PI) staining solution (10 μg/ml PI, 100 μg/ml RNAse/PBS) and analysed using a Cyan ADP Analyzer (Beckman Coulter) equipped with a 488 nm laser. Data was routinely collected from 10,000 cells and then analysed using FlowJo 7.6.3. Controls with no first antibody were used to set the threshold for counting phospho-T575 positive cells. For sorting cells that were later analysed by Western blotting live cells were labelled using the DNA stain H33342 (Sigma-Aldrich) and cells in G1 were sorted using a MoFlo Legacy cell sorter (Beckman Coulter) before being lysed and analysed.

### Mass spectrometry

Gel pieces were digested and desalted on a C18 packed pipette tip. Samples were injected onto an Ultimate 3000 nano HPLC (Dionex) system coupled to an Orbitrap mass spectrometer (Thermo Electron). Searches were carried out by Mascot and data were searched against IPI Human protein database. Data were analysed using the Central Proteomics Facility Pipeline (CPFP), which co-ordinates database searches carried out using the following search engines; Mascot, X! Tandem and OMSSA and combines search results as well as threshold them for 1% false discovery rate (FDR) from statistics calculated using iProphet. The mass spectrometry proteomics data have been deposited to the ProteomeXchange Consortium via the PRIDE [15] partner repository with the dataset identifier PXD006459.

### Statistical analysis

All experiments were carried out in 3 biological repeats except ChIP and flow cytometry studies which were performed on 4 biological repeats. Differences between the conditions in these studies were evaluated using two-sided unpaired *t* tests (using Graph Pad) and were considered significant when the probability value, *P*, was <0.05 (shown on graphs as *) and <0.01 (shown as **). Non-significant differences in all cases are labeled as NS. Where relevant, results are shown as a mean ± standard error. For image analysis (of immunofluorescence data) between 30 and 50 cells were analysed from each condition using Image J. Macro for Image J analysis presented in S1 Appendix. Oct-1 staining intensity per unit area of lamina ring (defined by lamin B1staining) compared to the Oct1 signal from rest of the nucleus (nucleoplasm). Two-sided paired t-test was applied to determine significance level (defined as above).

## Results

### Oct-1 association with lamin B1 is a dynamic process

**Fig 1:**
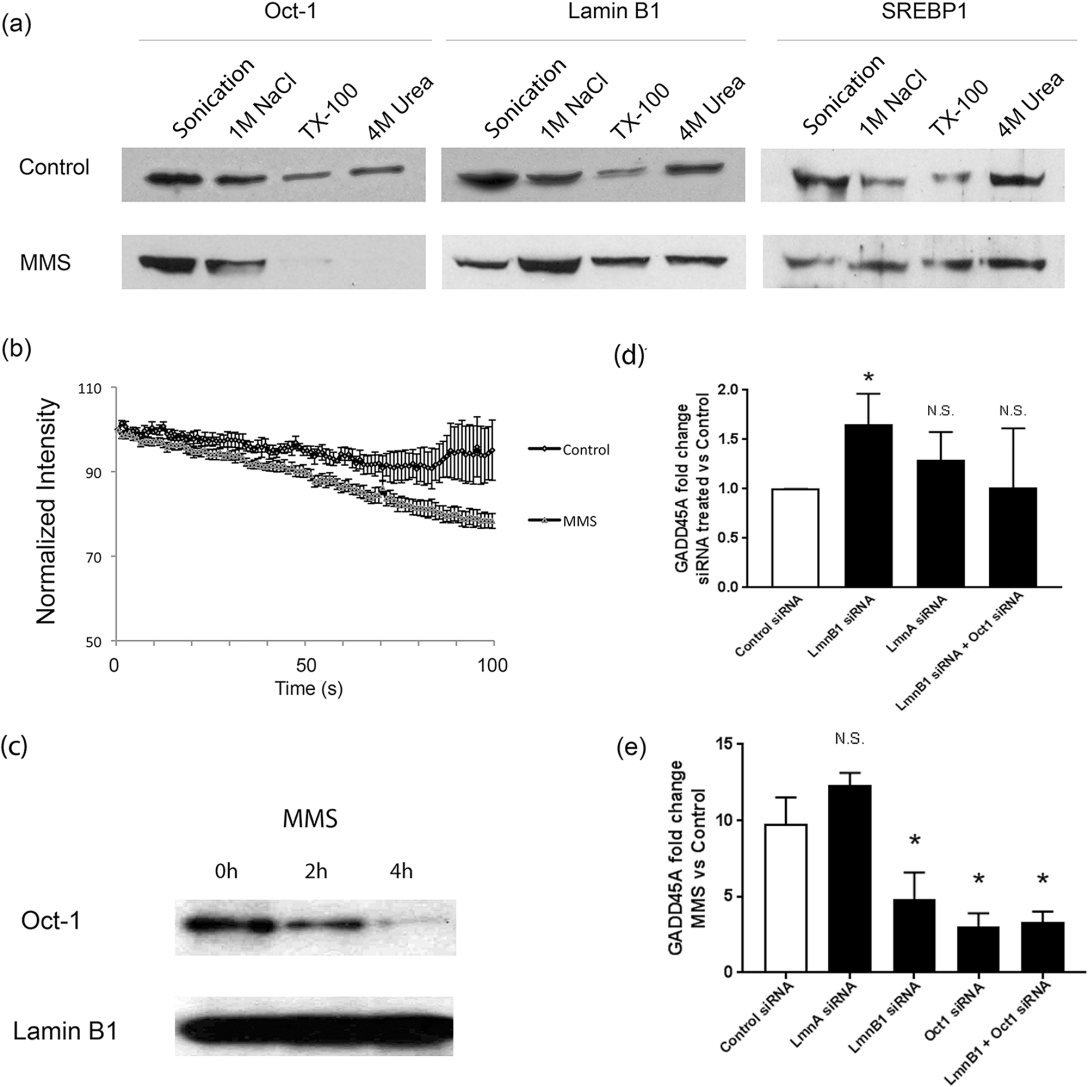
Oct-1 interaction with the nuclear lamina is dynamic. (a) Sequential nuclear extraction of Oct-1, lamin B1 and SREBP1 from control HeLa cells and cells treated with MMS. Oct-1 extractability is increased following MMS treatment showing reduced association within the nucleus following this treatment. (b). FLIP analysis of GFP-Oct-1 stability at the nuclear envelope: Normalised intensity measurements of regions of interest containing the peripheral GFP-Oct1 signal were used to assess fluorescence loss in photobleaching for control HeLa cells, or cells treated with MMS. The more rapid loss of GFP = Oct1 after MMS treatment confirms a weakened interaction with the nuclear periphery. (c) **Co-immunoprecipitation of Oct1 with lamin B1**: Control and MMS-treated HeLa cells were subject to immunoprecipitation using an anti-lamin B1 antibody. IP samples were then analysed using anti-lamin B1 and anti-Oct-1 antibodies. This shows a progressive loss of a direct interaction between lamin B1 and Oct1 following MMS treatment. (d) Expression of an Oct1 target gene is increased when lamin B1 expression is reduced: *GADD45A* expression was assessed using qPCR in HeLa cells transfected with control siRNA or siRNA directed against lamin A, lamin B1 or lamin B1 and Oct-1 together. Only lamin B1 siRNA transfection had a significant effect on *GADD45A* expression. (e) Stress mediated increase in an Oct1 target gene expression requires lamin B1 and Oct1: *GADD45A* expression was assessed in HeLa cells transfected with siRNA directed against lamin A, lamin B1, Oct-1 or lamin B1 and Oct-1 together, comparing MMS treatment with control. Transfection with the latter three combinations reduces the response to MMS treatment indicating the involvement of both lamin B1 and Oct-1 in the control of *GADD45A* expression following MMS treatment.

We have previously shown that Oct-1 sequestration at the NE is lost in the absence of full length lamin B1 in *Lmnb1*^Δ/Δ^ MEFs. In order to investigate whether this sequestration is constitutive or has a functional role as a response mechanism, we analysed lamin B1—Oct-1 interactions under conditions of genotoxic stress induced by MMS. We used sequential nuclear extraction which distinguishes different pools of proteins depending on their strength of association with the nuclear lamina and showed that, as in MEFs, Oct-1 is tightly associated with the insoluble lamina-containing nuclear fraction in HeLa cells since it persists even in the fraction remaining after 4 M urea extraction (Fig 1, section a). This tight association is lost within 2 hours of MMS treatment suggesting that the association of Oct-1 with the NE is dynamic. A control transcription factor, SREBP1, which is known to associate with lamin A and not lamin B1 [16] was used to show that the loss of Oct-1 sequestration as a result of MMS treatment is indeed specific and not a general feature of the nuclear lamina or its interacting partners (Fig 1, section a). Loss of Oct-1 sequestration following MMS treatment was also shown using a fluorescence loss in photobleaching (FLIP) approach in live cells expressing GFP-Oct-1. In control HeLa cells, 92% of GFP-Oct-1 showed stable association within the nucleus, while only 76% showed stable association after MMS treatment (Fig 1, section b; n = 10; p<0.01). Although the sequential extraction and FLIP experiments demonstrate that Oct-1 is less tightly associated within the nucleus upon MMS treatment they do not prove that this is due to a loss of direct interaction with lamin B1.

To address this issue, we immunoprecipitated lamin B1 using an anti-lamin B1 antibody and assessed the levels of co-immunoprecipitated Oct-1 using Western blotting. Oct-1 can be pulled down with lamin B1 using co-IP in control untreated cells but the amount is reduced in MMS treated cells (Fig 1, section c) demonstrating that the loss of sequestration is due to a loss of interaction with lamin B1.

One of the known functional consequences of the observed increased availability of Oct-1 following MMS treatment, is an elevation in the expression levels of one of its targets, *GADD45A*, in a p53-independent manner [17–19]. We wanted to investigate if the change in expression level of *GADD45A* following MMS treatment is a result of the loss of sequestration of Oct-1 by lamin B1. In order to do so we performed quantitative real-time PCR (qRT-PCR) analysis of *GADD45A* transcript levels following lamin B1 siRNA transfection. Reduction of lamin B1, but not lamin A, levels were found to result in an increased expression level of *GADD45A*. This increase in expression level was not observed when cells were transfected with a mixture of lamin B1 and Oct-1 siRNAs (Fig 1, section d). These results indicate that *GADD45A* expression is regulated by a pathway that involves both lamin B1 and Oct-1 and suggest that the increased expression of *GADD45A* in response to MMS might involve the same pathway. In order to test the latter, we assessed *GADD45A* expression levels following MMS treatment in cells that had been transfected with lamin B1 siRNA, Oct-1 siRNA or a combination of both. Reducing lamin B1 or Oct-1 levels or both prior to MMS treatment results in a similar reduced *GADD45A* expression response (Fig 1, section e), which supports the hypothesis that upon MMS treatment, Oct-1 sequestration by lamin B1 is lost and as a result Oct-1 becomes more readily available to bind its target sequences which results in an increased expression of *GADD45A*. We next wanted to identify the mechanism of Oct-1 release.

### Loss of Oct-1 sequestration is JNK phosphorylation-dependent

**Fig 2:**
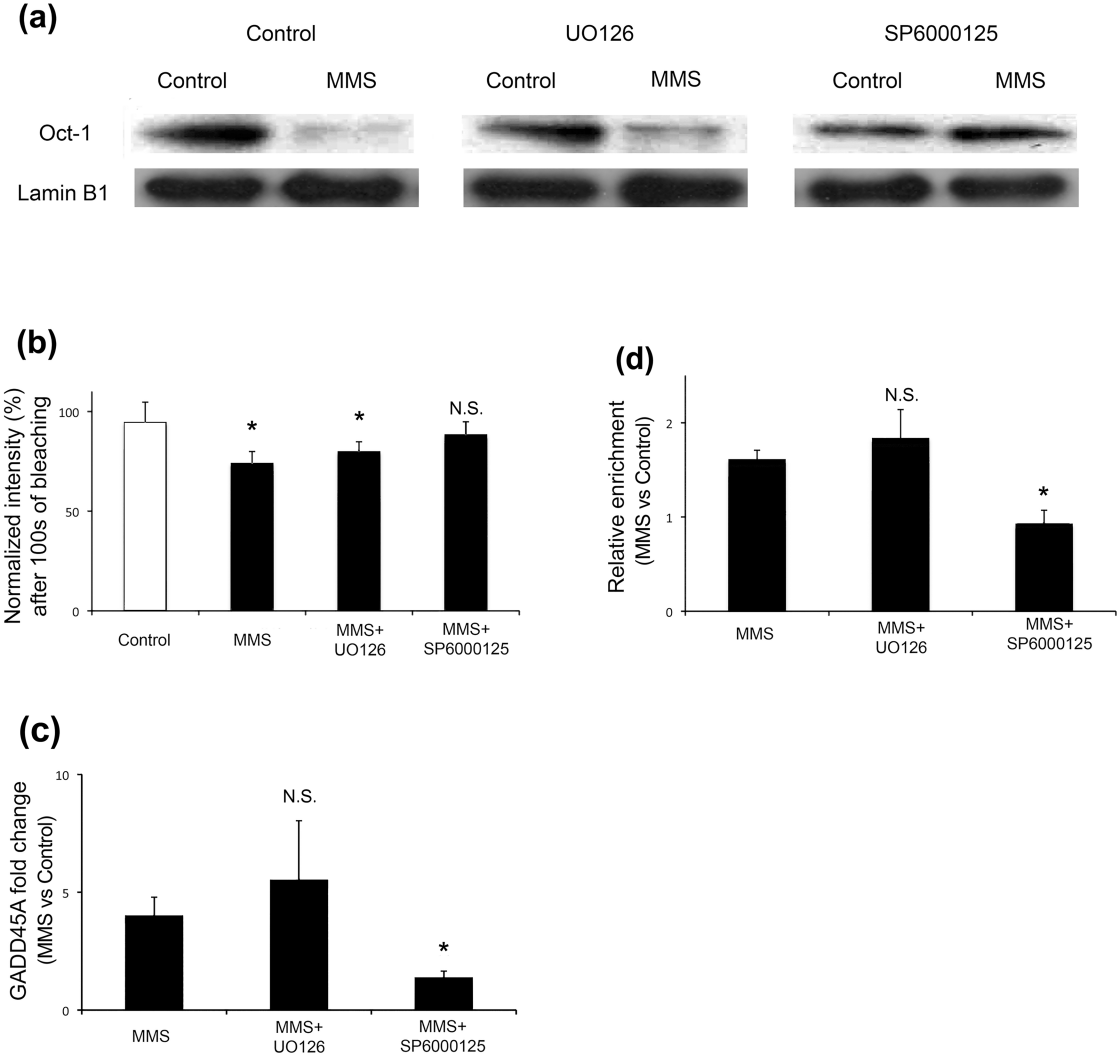
Loss of Oct-1 sequestration by lamin B1 in response to MMS is JNK dependent. (a) Co-immunoprecipitation of Oct1 with lamin B1: The lamin B1 Co-IP experiments shown in Fig 1 were repeated in HeLa cells that had been pre-treated with a JNK inhibitor (SP6000125) or ERK inhibitor (UO126) before control or MMS treatment. The JNK inhibitor reduces the MMS-dependent release of Oct-1 from lamin B1. (b) FLIP analysis of GFP-Oct-1 stability at the nuclear envelope: Normalised intensity measurements of regions of interest containing the peripheral GFP-Oct1 signal were used to assess fluorescence loss in photobleaching for control HeLa cells, or cells treated with MMS, with or without kinase inhibitor pretreatment. The graph displays the mean intensity value after multiple bleach cycles over 100 seconds, and confirms the effect of MMS shown in Fig 1 (section b). This weakened interaction effect is still seen after ERK inhibition, but is lost if JNK is inhibited. (c) MMS-induced GADD45A expression: *GADD45A* expression was assessed using qPCR in control and MMS treated HeLa cells that had received no pretreatment, or treatment with ERK or JNK inhibitors. The ratio of fold change in *GADD45A* transcript level for MMS over control is shown; only when JNK is inhibited is the MMS-induced elevation of GADD45A prevented. (d) Oct1 occupancy at GADD45A promoter site: The binding of Oct-1 to its target sequence within the promoter region of *GADD45A* was assessed using qPCR following Oct1 ChIP. The graph shows relative enrichment of Oct1 occupancy at the *GADD45A* promoter in MMS-treated versus control cells. Thus, a ratio >1 indicates higher occupancy after MMS treatment. Pretreatment with an ERK inhibitor had no effect, while inhibition of JNK abolished the MMS-induced increase in promoter site occupancy.

One of the ways by which protein-protein interactions are regulated is phosphorylation. MMS treatment is known to activate JNK [20, 21], making JNK a candidate kinase to be involved in the loss of Oct-1 sequestration. Under the conditions used in the current study, JNK was found to be activated after 2 hours of MMS treatment as detected by Western blotting to detect phosphorylated JNK (p-JNK) and phosphorylated c-Jun (p-c-Jun, downstream target of activated JNK) (S1 Fig). In order to investigate this, HeLa cells were pre-treated with either a JNK inhibitor (SP6000125) [22, 23] or an extracellular signal—regulated kinase (ERK) inhibitor (UO126) [24, 25]. Cells were then incubated with MMS as before and lamin B1 was immunoprecipitated. The amount of Oct-1 that was observed to co-IP with lamin B1 was reduced following MMS treatment in the case of the ERK inhibitor pre-treated cells (by 79% after 4 h in this representative experiment), which is similar to the control co-IP experiment (71%) (Fig 2, section a). When cells were pre-treated with the JNK inhibitor no reduction in the Oct-1 fraction was observed following co-IP (Fig 2, section a). This means that the loss of Oct-1- lamin B1 interaction when cells are treated with MMS is dependent at least in part on a phosphorylation event carried out by JNK, but not ERK. This finding is confirmed by FLIP of GFP-Oct-1 since pre-treating cells with the JNK inhibitor does not result in the reduced stability following MMS treatment observed in control cells or those pre-treated with the ERK inhibitor (Fig 2, section b).

**Fig 3:**
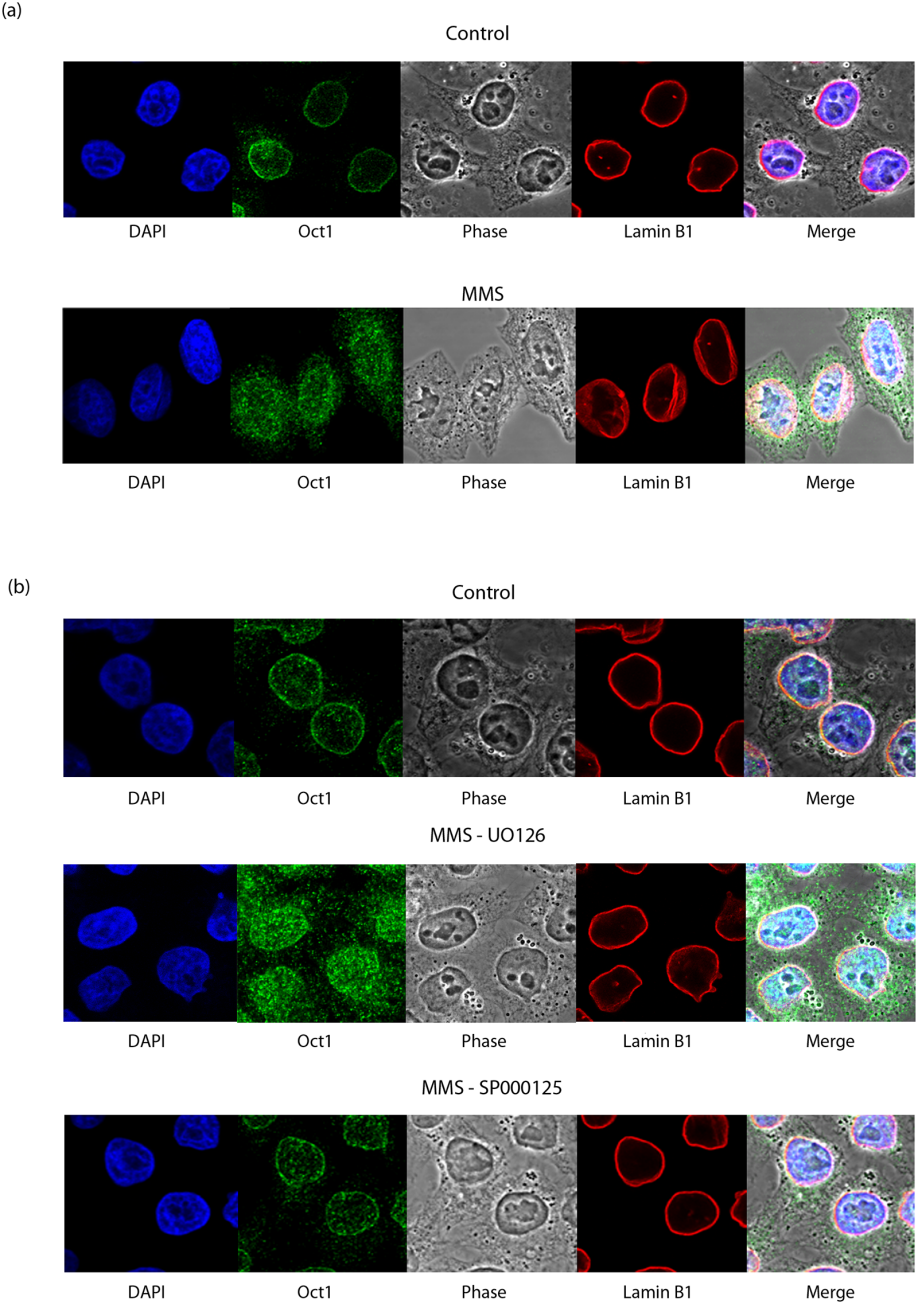
Loss of Oct-1 sequestration by lamin B1 in response to MMS is JNK dependent. **(a)** Direct evidence for JNK dependent release of Oct-1 1 from lamin B1 obtained by immunostaining with anti-Oct-1 and anti-lamin B1 antibodies after MMS treatment. Co-localisation of Oct-1 to nuclear lamina is lost after 4h MMS treatment. (b) The loss of peripheral Oct-1 sequestration caused by MMS treatment is absent when cells are pre-treated with JNK inhibitor (SP6000125), but not ERK inhibitor (UO126).

The downstream effects of Oct-1 release, i.e. binding of Oct-1 to its target sequence and the resulting increase in *GADD45A* expression were also tested using qRT-PCR (Fig 2, section c) and ChiP (Fig 2, section d) respectively. Fig 2 (section d) shows the ratio of *GADD45A* sequence detected in MMS versus control Oct-1-ChIP experiments; a ratio greater than 1 demonstrates increased Oct-1 binding at the *GADD45A* promoter site in MMS-treated versus control samples. MMS treatment results in increased Oct-1 binding to its target sequence in control and ERK inhibitor treated cells, but not in JNK inhibitor treated cells. As a result, *GADD45A* expression increases in the former two experimental conditions and not in the latter, as shown by measuring the fold change between untreated and MMS-treated samples using *GADD45A*-specific qRT-PCR (Fig 2, section d). Further evidence supporting involvement of JNK kinase in regulation of Oct-1 release from lamin B1 was obtained by immunostaining Oct-1 and lamin B1 in control and MMS treated HeLa cells (Fig 3). In agreement with a lamin B1 role in Oct-1 sequestration, untreated HeLa cells show localisation of Oct-1 around the nuclear periphery, overlapping with anti-lamin B1 immunostaining. This co-localisation is lost after 4 h treatment with MMS (Fig 3, section a), which induces release of Oct-1 from the nuclear periphery so it is then detected diffusely throughout the nucleus. These differences of Oct 1 distribution in nuclei of control and MMS treated cells are highly significant (S3 Fig). The same pattern of staining was observed in HeLa cells treated with both MMS and ERK inhibitor. However, when JNK inhibitor is added instead of ERK inhibitor, lamina co-localisation of Oct-1 is not altered by MMS treatment (Fig 3, section b and S3 Fig) and this transcription factor remains bound to nuclear lamina. Taken together these results indicate that loss of Oct-1 sequestration and its functional downstream consequences are all dependent, at least in part, on a JNK phosphorylation event, although they do not identify the phosphorylation target.

### Involvement of the phosphorylation of lamin B1 T575 in Oct-1 release

We considered lamin B1 as a possible phosphorylation target to explain the observations made above and took two approaches to testing this hypothesis. In the first approach, we immunoprecipitated lamin B1 from cells with and without MMS treatment, and subjected the isolated protein to enzymatic fragmentation (using either trypsin, or elastase and endo-Lys-C in combination to ensure optimal fragmentation, and the best sequence coverage) followed by mass spectrometry (S2 Fig). We analysed the MS/MS data using a custom software pipeline (as described in Materials and methods), with phosphate addition explicitly included as a permitted post-translational modification in the peptide searches. In this way, all phosphorylation events could be ascribed to specific residue positions in the sequenced peptides.

**Fig 4:**
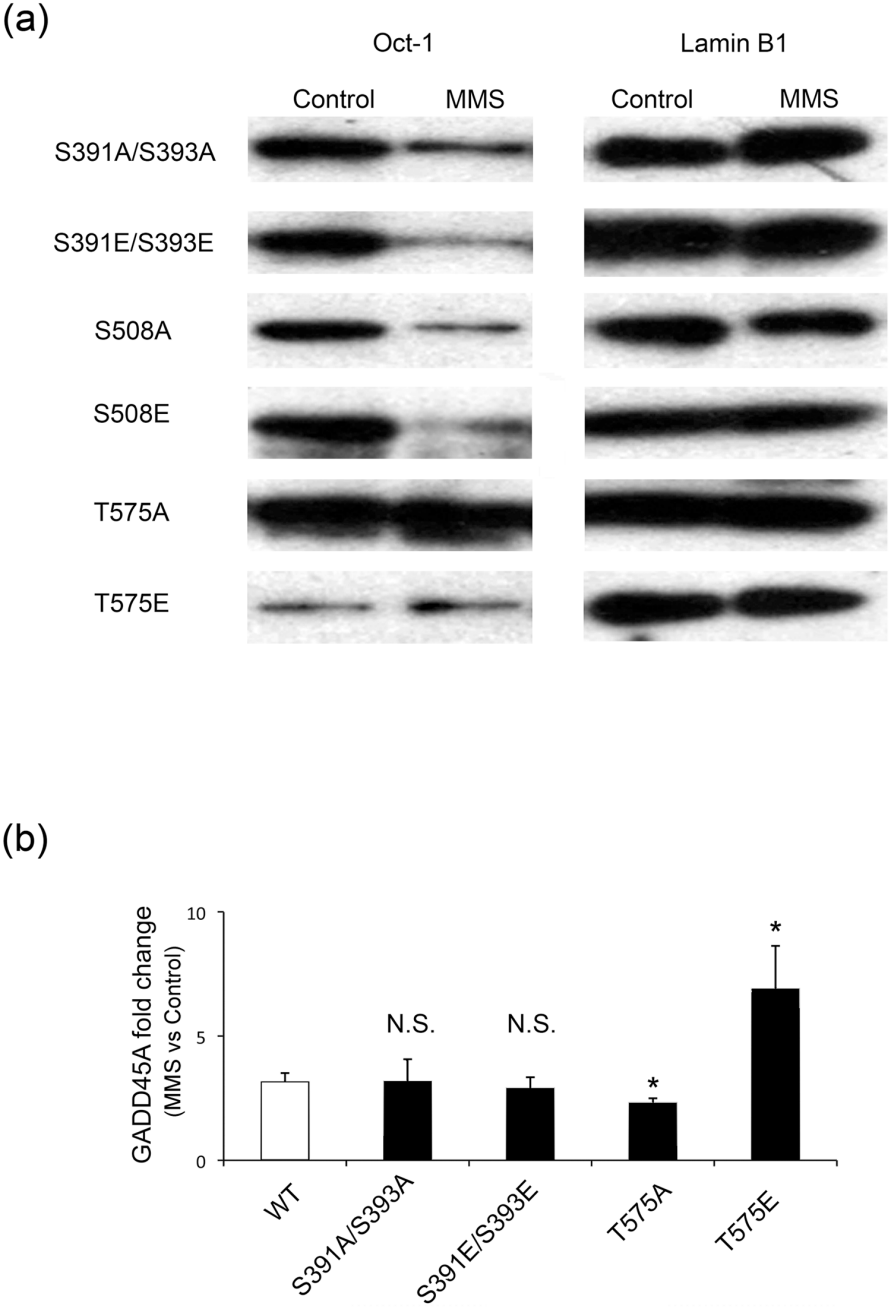
Phosphorylation of residue Threonine-575 within lamin B1 is responsible for the release of Oct-1 from lamin B1 following MMS treatment. (a) Co-immunoprecipitation of Oct-1 with mutant lamin B1: Using site directed mutagenesis, several putative phosphorylation sites within the C-terminus of lamin B1 were mutated to either an alanine or a glutamic acid residue. HeLa cells were transfected to express a GFP-tagged version of each of the mutants and subject to MMS treatment followed by anti-GFP IP. GFP-Lamin B1 precipitation controls (right panel) and co-immunoprecipitated Oct-1 (left panel) are shown. Reduced pull-down of Oct-1 after MMS is seen for all mutations except those at threonine 575. Note that the mutation mimicking constitutive phosphorylation (T575E) binds very little Oct-1 under any condition, while the mutation that cannot undergo phosphorylation (T575A) binds Oct-1 strongly even after MMS treatment. (b) MMS-induced GADD45A expression: *GADD45A* expression was assessed using qPCR in control and MMS treated HeLa cells that had been transfected to express the mutant GFP-lamin B1 proteins. The ratio of fold change in *GADD45A* transcript level for MMS over control is shown; only mutations at T575 change the behaviour of GADD45A transcript level. The T575A mutant sequesters Oct-1 even after MMS treatment, resulting in a reduced GADD45A induction, whereas the T575E mutant, which is unable to bind Oct-1 effectively, permits an enhanced response to MMS.

This approach identified threonine 575 (T575) near the C-terminus of lamin B1 as a site of MMS-dependent selective phosphorylation. Our second approach was to identify potential phosphorylation sites within lamin B1 and focus on four potential residues; S391, S393, S508 and T575. All of these residues are absent from the severely truncated lamin B1 fragment present in the *Lmnb1*^Δ/Δ^ cells that lack Oct1 sequestration [12]. Using site-directed mutagenesis we generated full-length GFP-lamin B1 alanine (A) and glutamic acid (E) mutants of these sites, which would mimic a state of non-phosphorylation and constitutive phosphorylation respectively. Cells were transfected with these constructs 24 hours prior to MMS treatment. IP using an anti-GFP antibody was then performed in control and MMS treated cells, to capture only the mutant lamin B1 protein and not the wild type protein also present in the cells. Reduced levels of Oct-1 were pulled down with lamin B1 following MMS exposure (as seen in the experiment shown in Fig 1) in the case of the S391A/S393A, S391E/S393E, S508A and S508E mutants (Fig 4, section a). Thus, the MMS-dependent reduction in lamin B1-Oct-1 interactions seen in wild type protein persisted for these mutations, suggesting that these phosphorylation sites are not involved in the regulation of Oct-1 lamin B1 interaction and Oct-1 release.

The T575A mutant however showed a different pattern. Oct-1 was pulled down with the T575A mutant in the untreated sample and no loss of co-IP was observed after MMS treatment. In the case of the T575E mutant, very little Oct-1 was pulled down both in control and MMS treated sample (Fig 4, section a). This indicates that the Oct-1 lamin B1 interaction and its regulation are dependent on phosphorylation of T575 of lamin B1.

Quantitation of the expression of *GADD45A* using qRT-PCR was used to further support this conclusion (Fig 4, section b). The bar chart shows the fold change (MMS-treated/untreated) in *GADD45A* transcript level seen in cells transfected with wild type or lamin B1 mutants. The MMS-driven increase in expression of GADD45A is seen in cells transfected with S391A/S393A, and S391E/S393E mutants or wild type lamin B1. However, transfection of cells with the T575A mutant resulted in a significant reduction in the increase of *GADD45A* expression seen following MMS treatment, while transfection with the T575E mutant resulted in a more elevated response. Since the transfected cells retain endogenous lamin B1 expression, these results suggest a *trans* dominant effect of co-expression of the T575 mutant proteins. This is probably due to the glutamic acid mutant not being able to sequester Oct-1, leaving more Oct-1 available to elicit the *GADD45A* response, while the alanine mutant cannot release Oct-1 and hence less Oct-1 is made available in the nucleoplasm to impact expression of *GADD45A*. There is still some increase in the expression level, however, and this is probably due to the presence of endogenous copies of wild type lamin B1 that are still able to bind and release Oct-1. We note that these observations are not contradictory to our data showing reduced GADD45 response after lamin B1 siRNA treatment (Fig 1, section e), because the latter shows differences in expression of GADD45 after MMS treatment in the situation when total lamin B1 levels are already depleted by siRNA treatment.

### Detection of endogenous phospho-T575 lamin B1

We next generated a rabbit polyclonal antibody that recognises phosphorylated T575 (anti-p-lamin B1) in order to use the antibody to confirm that phosphorylated T575 occurs on endogenous lamin B1 and is indeed involved in the cellular response to MMS treatment and the downstream consequences. The resulting antibody was lamin specific, phospho-selective and compatible with both immunofluorescence and western blotting (S4 Fig). However, we swiftly found that T575 is heavily phosphorylated in a cell cycle dependent manner and specifically during mitosis and hence there was some difficulty in analysing phosphorylation following MMS treatment. The anti-p-lamin B1 signal in western blotting was strongly diminished following a double thymidine block to arrest the cells at the G1/S boundary (section a); the signal gradually increased after release from the block. There was also a dramatic increase in the anti-p-lamin B1 signal following mitotic arrest using nocodazole, suggesting that this site is phosphorylated prior to the mitotic spindle checkpoint (section b). Furthermore, immuno-fluorescence clearly shows an increase in signal in mitotic cells (section c).

**Fig 5:**
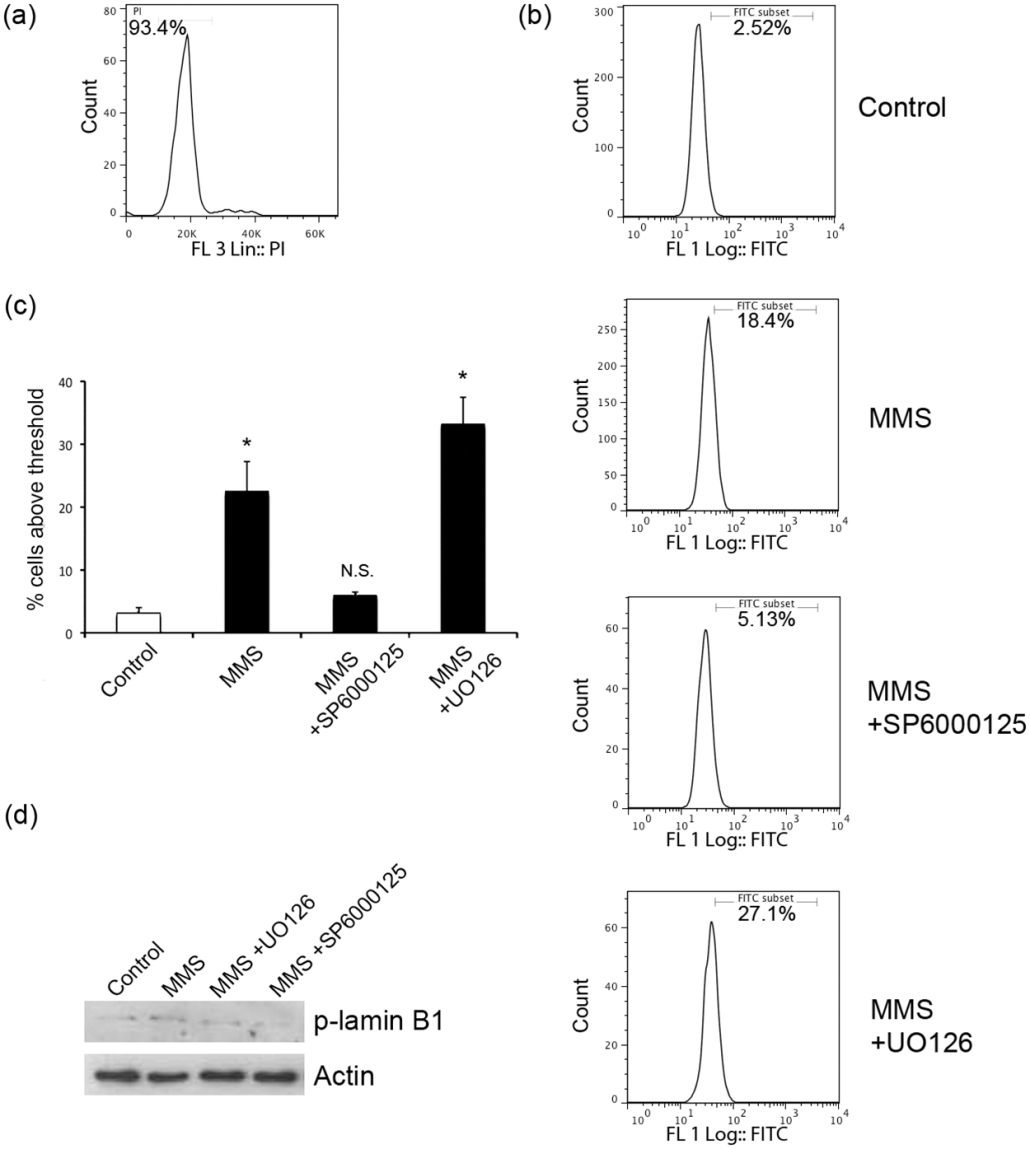
Analysis of lamin B1 phosphorylation on T575. Human dermal fibroblasts (HDFs) were incubated under the indicated conditions before being fixed and labelled with anti-p-lamin B1 and propidium iodide (PI) to analyse T575 phosphorylation and cell cycle stages respectively. (a) Flow cytometry analysis of cell cycle phases: A representative cell cycle profile based on PI staining is shown. Only G1 cells (left peak with 2N DNA content) were analysed for p-lamin-B1 signals shown in all subsequent analyses. (b) Flow cytometry analysis of G1 interphase Phospho T575 signals: Anti-pT575 signals in the G1-gated population are shown for control cells as well as those treated with MMS, or MMS and kinase inhibitors as indicated. (c) Quantitation of interphase cells with pT575 signal: Summary of percentage of cells above T575 phosphorylation threshold set in (b). Controls without first antibody were used to set the threshold for the pT575 signal gate. There is a significant difference between control and MMS treated cells, and this is also seen in the case of ERK inhibitor (p<0.01) pre-treatment, but no significant difference in the case of the JNK inhibitor pre-treatment (p = 0.06). Data shown is mean +/- s.e.m. (d) Western blotting of G1-gated interphase cells: Sorted G1 cells were lysed and analysed by Western blotting using the anti-p-T575 antibody. The results confirm those observed by flow cytometry. Detection of actin is used to demonstrate equal loading.

To overcome the problem of high level mitotic phosphorylation at this site without perturbing cells by chemical cell cycle synchronisation, we used flow cytometry as an approach to analyse the phosphorylation of T575 following MMS treatment. Furthermore, we used primary cells, human dermal fibroblasts (HDFs) to demonstrate that the effects we are seeing are not exclusive to transformed (HeLa) cells. HDF cells were fixed and labelled with anti-p-lamin B1 and the quantitative DNA dye propidium iodide (PI) in order to analyse both the phosphorylation state and cell cycle stage under different conditions and assess the phosphorylation status of lamin B1 in separate stages of the cell cycle. Phosphorylation of lamin B1 at T575 was elevated in G1 cells following MMS treatment (Fig 5, sections a and b). Using this flow cytometry approach we found that pre-treating cells with the JNK inhibitor, but not the ERK inhibitor, prevented a significant increase in phosphorylation in response to MMS (Fig 5, section c). As a final biochemical demonstration that interphase cells contain phospho-T575 lamin B1 we physically sorted cells based on their DNA content to collect G1 cells only. Using these purified G1 populations for Western blotting enabled us to show that phospho-T575 lamin B1 is detectable in interphase cells (Fig 5, section d). Furthermore, by purifying G1 cells from MMS and kinase inhibitor treated populations, this approach enabled us to confirm that MMS stress increases the interphase phospho-T575 lamin B1 signal in a JNK-dependent manner (Fig 5, section d). Flow cytometry experiments with JNK and ERK inhibitors were repeated in HeLa cells and similar results obtained reconfirming phosphorylation of T575 lamin B1 by JNK kinase (S5 Fig).

T575 phosphorylation in HeLa cells was also assessed following various other stress conditions. We found that heat shock and hydrogen peroxide treatment cause a significant increase in T575 phosphorylation, while zeocin did not (S6 Fig, section a). Effect similar to MMS treatment (on Oct-1 release from lamin B1) was also evident on immunostaining of control, heat shock and hydrogen peroxide treated HeLa cells (sections b and c). Localisation of Oct-1 staining at the nuclear periphery is lost after 10 min heat shock or 4 h hydrogen peroxide treatments. This demonstrates that interphase lamin B1 phosphorylation is not an MMS-specific response, but extends to some other types of cellular stressors.

**Fig 6:**
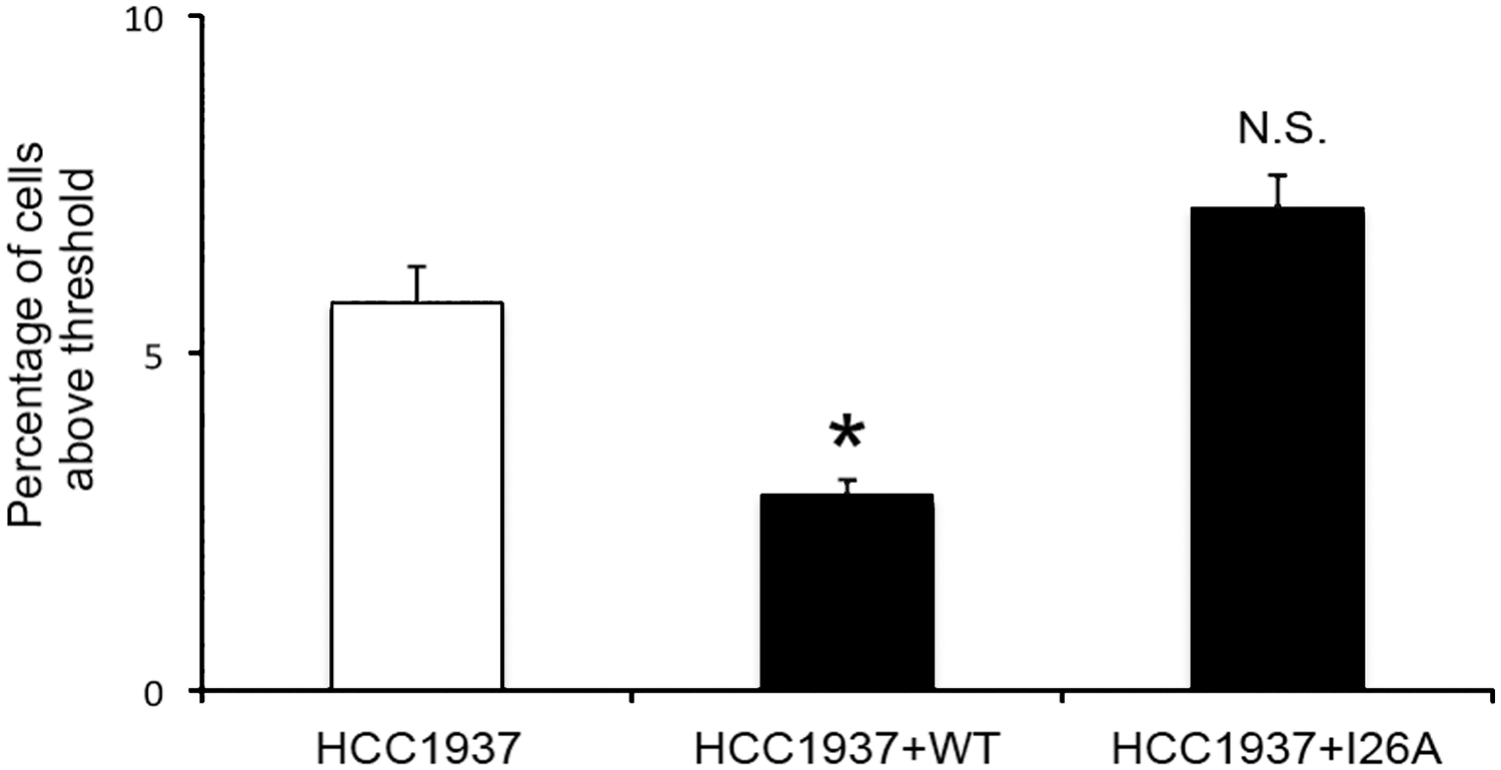
Analysis of T575 phosphorylation in human breast cancer cells. HCC1937, HCC1937 cells transfected to express full length functional BRCA1 (HCC1937+WT) and HCC1937 cells transfected to express BRCA1 harbouring a ubiquitin ligase-inactivating I26A mutation (HCC1937+I26A) were analysed for T575 phosphorylation by flow cytometry as described. There is a significant reduction in phosphorylation when WT BRCA1 function is restored in HCC1937 cells (p<0.05) but not when the cells express an inactive I26A BRCA1 (p = 0.1). Values represent mean +/- SEM of the percentage of cells with pT575 signal above a threshold determined by first antibody deletion controls.

In order to examine a system undergoing chronic pathophysiological stress, we also investigated T575 phosphorylation in HCC1937 cells, human breast cancer cells bearing two loss of function alleles of BRCA1, a gene encoding a large E3 ubiquitin ligase involved in DNA damage repair [26, 27]. The ubiquitin ligase activity of BRCA1 has been reported to contribute to its DNA damage repair function [28–30] We compared HCC1937 cells which exhibit a severe deficit in BRCA1-mediated DNA repair, [31, 32] with cells that were rescued with full length BRCA1 (HCC1937+WT) or transfected with a full-length, point-mutated form of BRCA1 which lacks its ubiquitin ligase activity (HCC1937+I26A) (Fig 6). We found that T575 phosphorylation of G1 cells was significantly higher in HCC1937 cells compared to those that had been transfected with full length WT BRCA1 (HCC1937+WT; p<0.05). In contrast, there was no significant difference in T575 phosphorylation between HCC1937 cells and those transfected with the I26A mutant form of BRCA1 (HCC1937+I26A; p = 0.1), although we note a trend for the I26A mutant protein to cause a worsening of the defect, as was also seen in the context of breast cancer cell motility [33]. Our result suggests that T575 phosphorylation is relevant to stress caused by mutations in genes that are involved in DNA damage repair as well as cell stress caused by chemical treatments.

## Discussion

We have previously shown that lamin B1 can bind to and sequester Oct-1 at the NE. There was no evidence at that time, however, that this interaction had any biological functional significance since the loss of sequestration was observed only in mutant MEFs lacking full length lamin B1. In the current study we wanted to investigate whether this interaction was dynamic and regulated, or static. We used cellular stress as a trigger to investigate the nature of Oct-1 interaction with full length lamin B1.

Methyl-methane-sulfonate (MMS) is a genotoxic agent that causes DNA alkylation [34]. There have been previous reports of elevated Oct-1 protein levels in MMS treated cells, without a change in transcript level, in a number of human cell types including HeLa, MCF-7 and H1299 [35]. This response starts as early as 30 min after treatment and the levels of Oct-1 return to normal levels within 24 hours suggesting that the response is reversible. We therefore hypothesised that this elevation in extractable Oct-1 could be due to a controlled loss of sequestration by lamin B1.

We found that, following MMS treatment, Oct-1 was released from the NE and that this was due to loss of a direct interaction with lamin B1. This release resulted in an increase in the expression of *GADD45A* in a manner that is dependent on Oct-1 and lamin B1 but not lamin A. Using a combination of MMS and kinase inhibitors we then identified that the loss of sequestration following MMS treatment and the consequent increase in *GADD45A* expression were dependent on JNK. This is consistent with previous reports that have identified JNK as being activated following MMS treatment [21] and JNK kinase phosphorylation of targets being a common response to oxidative stress [36]. Using mass spectrometry and site directed mutagenesis followed by IP we then demonstrated that threonine 575 of lamin B1 is a target for this stress-induced phosphorylation. We generated a phospho-specific antibody directed against phospho-T575 and used it to confirm the above results. We subsequently found that the antibody readily detects mitotic phosphorylation of this residue in lamin B1. In order to examine interphase T575 phosphorylation, we turned to DNA labelling and flow cytometry to isolate cells in the G1 phase of the cell cycle. This enabled us to confirm that the interphase phosphorylation event results from stress and is JNK-dependent.

Phosphorylation of T575 occurs on a large scale during mitosis (S4 Fig), consistent with a role (together with additional mitotic phosphorylation sites on lamin B1 as well as other lamins) in regulation of nuclear disassembly for open mitosis. Phosphorylation of a much smaller proportion of lamin B1 at T575 would be enough to release sufficient Oct-1 to respond to stress. It is possible that released Oct-1 can also be localised to specific nuclear areas if the lamin B1 phosphorylation by JNK is focal and release occurs from copies of lamin occupying specific sites, such as areas of high negative curvature at the tips of nuclear invaginations [37]. We believe that this explains why we do not observe a massive increase in phospho-T575 signals in asynchronously growing cells using the antibody generated in the current study.

The NE and its components have previously been implicated in stress responses [6]. Lamin B1 levels are up-regulated and are thought to contribute to recovery from heat shock [37–39]. A group of antioxidant enzymes including glutathione transferase and catalase have been reported to associate with the outer nuclear membrane upon oxidative stress to form the so-called nuclear shield which increases their local concentration by nearly seven fold [40]. CCTα, which is an enzyme that associates with the NE and promotes nucleoplasmic reticulum proliferation, redistributes from the nucleoplasm to form foci which associate with lamin A/C under conditions of osmotic stress [41]. NE components are also thought to be important under conditions of genotoxic stress since cells from patients with laminopathies exhibit defects in DNA damage responses [42–44]. Cells from HGPS patients for example show delayed recruitment of 53BP1 to γH2AX DNA repair foci [45]. The exact roles and mechanisms by which NE components contribute to stress responses remain unknown, although phosphorylation of interacting partner proteins has been observed. For example, Oct-1 that regulates basal expression by recruiting and activating Pol II [46] is modified in multiple ways including O-GlcNacylation, ubiquitilation and phosphorylation at multiple sites [47, 48]. These phosphorylation events are involved in signaling pathways during the cell cycle and under cellular stress and sometimes affect the DNA binding characteristic of Oct-1 [49–52] or may induce Oct-1 pausing effect in order to poise genes for rapid transcription [46, 48]. Our data suggests an added level of control to such signaling events by the binding and release of Oct-1 from the NE through phosphorylation of its peripheral binding partner, lamin B1. More generally, our present study demonstrates that through phosphorylation of specific sites the nuclear lamina can provide a platform for controlled transcription factor sequestration and release in response to cellular stress conditions. Although lamin A/C phosphorylation has been previously implicated in signalling events [53–55], B-type lamin phosphorylation has only been implicated in nuclear disassembly during mitosis and apoptosis [56, 57]. Our findings are therefore the first to suggest that interphase phosphorylation of lamin B1 is involved in signalling as a result of cellular stress.

## Conclusion

**Fig 7:**
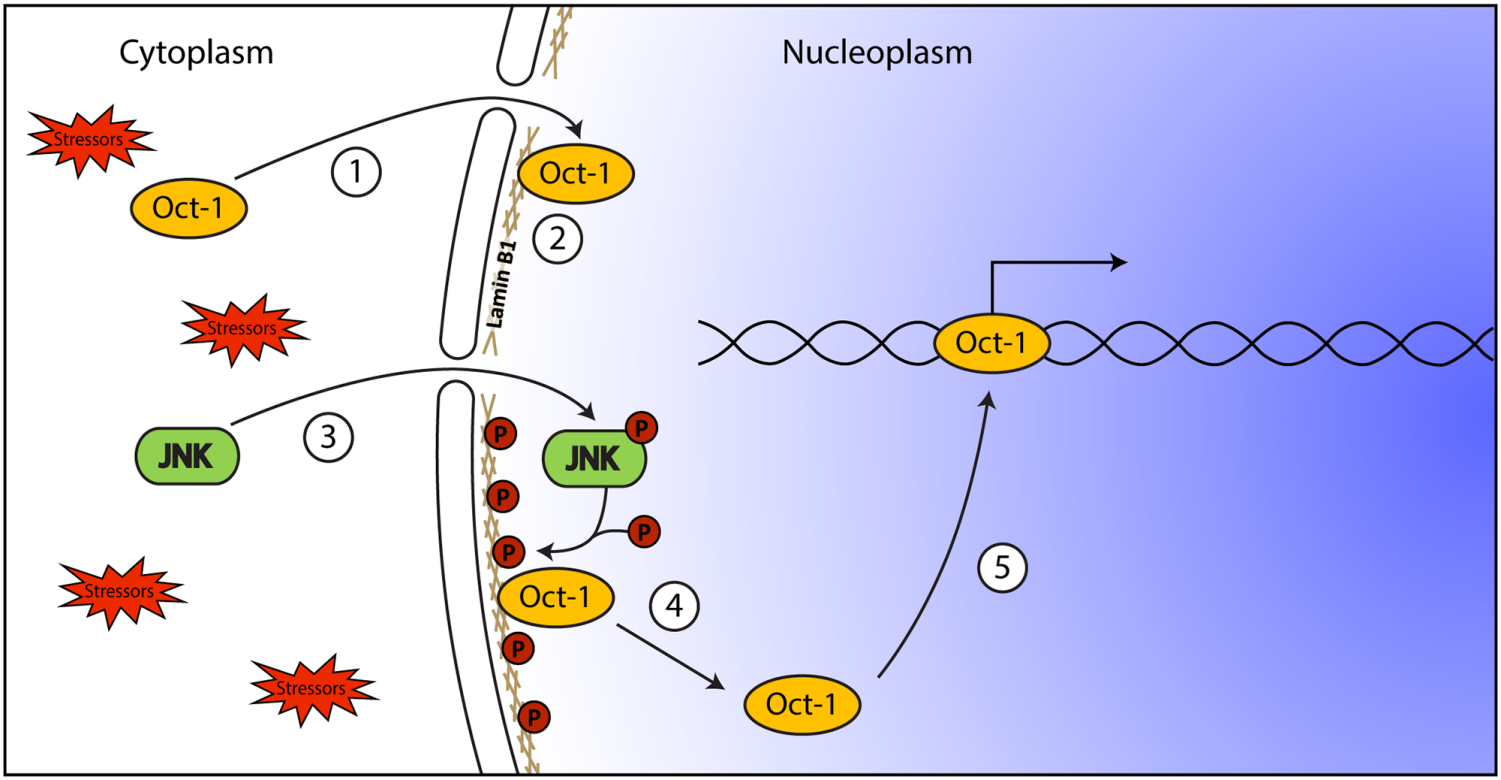
Schematic summary of main findings. Oct-1 that is imported into the nucleus (1) is tethered to the NE by interacting with lamin B1 (2). When cells are subjected to stress (e.g. MMS treatment), JNK is activated by phosphorylation (3) and in turn phosphorylates lamin B1 (4). The phosphorylation of lamin B1 results in release of Oct-1 from the NE and its binding to target sequences (5), regulating gene expression. Note that this generates a Boolean AND gate in which two events are required for full signalling, although they may occur independently in time and indeed in any order.

We note that the signalling pathway we describe creates a Boolean AND gate for cellular responses to specific stressful stimuli, in which two events are required for a full response (Fig 7). The events do not have to occur simultaneously, nor do they have to occur in a particular order; furthermore, such paired signals enable cells to respond in a modulated and potentially spatially restricted manner to stressors. In particular, the interphase phosphorylation status of lamin proteins may create a persistent epigenetic code that has the advantage of being erased each cell cycle during the mass lamin phosphorylation-dephosphorylation process that flanks mitosis.

## Supporting information

S1 Fig MMS treatment activates JNK. (a) Cells were treated with MMS as described and analysed by Western blotting to assess the activation of JNK using anti-p-JNK and subsequent modification of the target protein using anti-p-c-Jun. An anti-JNK antibody was used to verify equal loading. Results indicate that JNK is activated to p-JNK within 2 hours of MMS treatment, leading to c-Jun phosphorylation. (b, c) Levels of p-JNK in control and MMS treated cells were also assessed using flow cytometry. In (b) the left hand panels show propidium iodide staining of DNA to confirm that the treatment had not altered the cell cycle distribution of the population. The right hand panels show labelling of these cell populations with anti-p-JNK after control or MMS treatment. Note that the very robust Western blot signals in this well-validated pathway translate to small changes in the cytometry profiles; nonetheless, these changes are based on the measurement of labelling levels in 10,000 cells/profile and are highly significant. In (c) cells are first gated for G1 DNA content (from the propidium iodide channel on the x-axis) and then assessed for p-JNK content by counting percentage of cells above a threshold (on the y-axis) defined by a first antibody deletion control.
(TIF)

S2 Fig Sequence coverage of lamin B1 from the phospho-proteomic analysis showing residue (T575) that is selectively phosphorylated in MMS treated cells confirmed by mass spectrometry data. The sequence coverage of sequenced peptides (illustrated in bold) for lamin B1 is shown for the untreated control sample and the MMS treated sample. MS/MS fragmentation of lamin B1 peptide AGVVVEEELFHQQGTPRAS from the control sample shows no mass shift at threonine 575, whereas the MMS-treated sample contains the same peptide but with a phosphorylation of threonine 575.
(TIF)

S3 Fig Analysis of Oct-1 nuclear distribution in control and MMS treated cells: Effect of kinase inhibitors. Immunofluorescence microscopy images of individual nuclei (>30 per treatment) were analysed for the distribution of Oct-1 signal intensity per unit area using or Image J. Oct-1 at the nuclear lamina area (defined by lamin B1 staining) was compared to the internal nucleoplasmic Oct1 signal by taking a ratio of the two area-normalized intensity values. Differences in Oct-1 localization at the lamina ring and in nucleoplasm are highly significant (p<0.01) in the case of control (untreated) cells (top left). However, this difference was lost after MMS treatment led to loss of peripheral Oct1 sequestration (top right). Pretreatment of the cells with ERK inhibitor UO126 did not affect this response to MMS (lower left). In contrast, pretreatment with the JNK inhibitor SP000125 abolished the release of peripheral Oct1, leaving a significant difference between peripheral and nucleoplasmic signals (lower right). This further confirms the role of JNK kinase in Oct-1 release from nuclear lamina upon MMS induced stress.
(TIF)

S4 Fig An affinity purified anti-phospho-T575 lamin B1 antibody confirms phosphorylation of T575 of lamin B1 is regulated during the cell cycle. An affinity purified rabbit polyclonal anti-phospho-peptide antibody was raised against phospho-threonine 575 in lamin B1 and used to analyse phospho-T575 levels in control cells (a; lane C) and at intervals after releasing cells from a thymidine block (a) or after nocodazole treatment (b) indicating that T575 is phosphorylated during mitosis. The blot in (b) is of a construct of the 40 C-terminal amino acids of lamin B1 transfected into HeLa cells. (c) Fluorescent staining of unsynchronised HeLa cells with rabbit anti-pT575 (left panel), DAPI (centre panel) and anti- lamin B1 (right panel) showing intense phospho-T575 signals in mitotic cells. (d) ELISA confirmation of the selectivity of the affinity purified anti-phosphopeptide antibody against phospho-T575 lamin B1; control peptide (HQQGTPRASNRSC) has the same sequence as phosphopeptide (HQQG[Tp]PRASNRSC), but lacks phosphorylation at threonine T575.
(TIF)

S5 Fig Analysis of lamin B1 phosphorylation on T575 using flow cytometry. HeLa cells were incubated under the indicated conditions before being fixed and labelled with anti-phospho-lamin B1 and propidium iodide (PI) in order to analyse T575 phosphorylation and cell cycle stages respectively. (a) Cell cycle profiles based on PI staining; note the large G1 peak to the left, the G2/M peak to the right, and the intermediate saddle area of cells in S phase with intermediate DNA content. (b) G1 cells were then analysed for T575 phosphorylation levels under different treatment conditions. Data shown is a representative of at least four replicates. The threshold intensity (y-axis) for counting a cell as pT575 positive was determined as the lowest intensity value that excluded all cells in a first antibody deleted control. (There is a significant difference between control and MMS treated cells in the case of ERK inhibitor (p<0.05) and DMSO vehicle control (p<0.01) pre-treatment, but no significant difference in the case of the JNK inhibitor pre-treatment (p = 0.48). Data shown is mean +/- s.e.m.
(TIF)

S6 Fig Effects of the different cell treatments on Oct1 release form nuclear lamina. (a) Flow cytometry analysis of interphase (G1) phosphorylation events after various cell treatments. Summary of percentage of HeLa cells above T575 phosphorylation threshold (set as shown in S5 Fig and described in the legend). Heat shock and H_2_O_2_ treatments cause significant increase in interphase (G1) T575 phosphorylation, while zeocin treatments does not. (b) Microscopy evidence for the release of Oct-1 from lamin B1 after heat shock or H_2_O_2_ treatment. Co-localization of Oct-1 and lamin B1 at the NE is lost as shown by immunostaining using anti-Oct-1 and anti-lamin B1 antibodies. (c) Quantitation of experiments shown in panel (b) using Image J as described in the legend to S3 Fig.
(TIF)

S1 Table Real time-PCR and ChIP primers. (DOCX)

S2 Table GADD45A expression change in siRNA treated cells. Data for Fig 1 (section d).
(DOCX)

S3 Table GADD45A expression change in siRNA and MMS treated cells. Data for Fig 1 (section e).
(DOCX)

S4 Table GADD45A expression change in MMS and kinase inhibitors treated cells. Data for Fig 2 (section c).
(DOCX)

S5 Table GADD45A expression change in MMS treated mutated cells. Data for Fig 4 (section b).
(DOCX)

S6 Table Relative enrichment of GADD45A promoter occupancy in MMS and kinase inhibitors treated cells. Data for Fig 2 (section d). Part a. Relative enrichment of GADD45A promoter occupancy in MMS only treated cells. Part b. Relative enrichment of GADD45A promoter occupancy in MMS plus UO126 treated cells. Part c. Relative enrichment of GADD45A promoter occupancy in MMS plus SP000125 treated cells.
(DOCX)

S7 Table Site directed mutagenesis primer sequences. (DOCX)

S1 Appendix Macro for ImageJ analysis. (DOCX)
