# Supplementary figures and images for: Stress-induced release of Oct-1 from the nuclear envelope is mediated by JNK phosphorylation of lamin B1
Source: PLoS One. 2017 May 24;12(5):e0177990. doi: 10.1371/journal.pone.0177990 (PMC5443517; doi:10.1371/journal.pone.0177990)

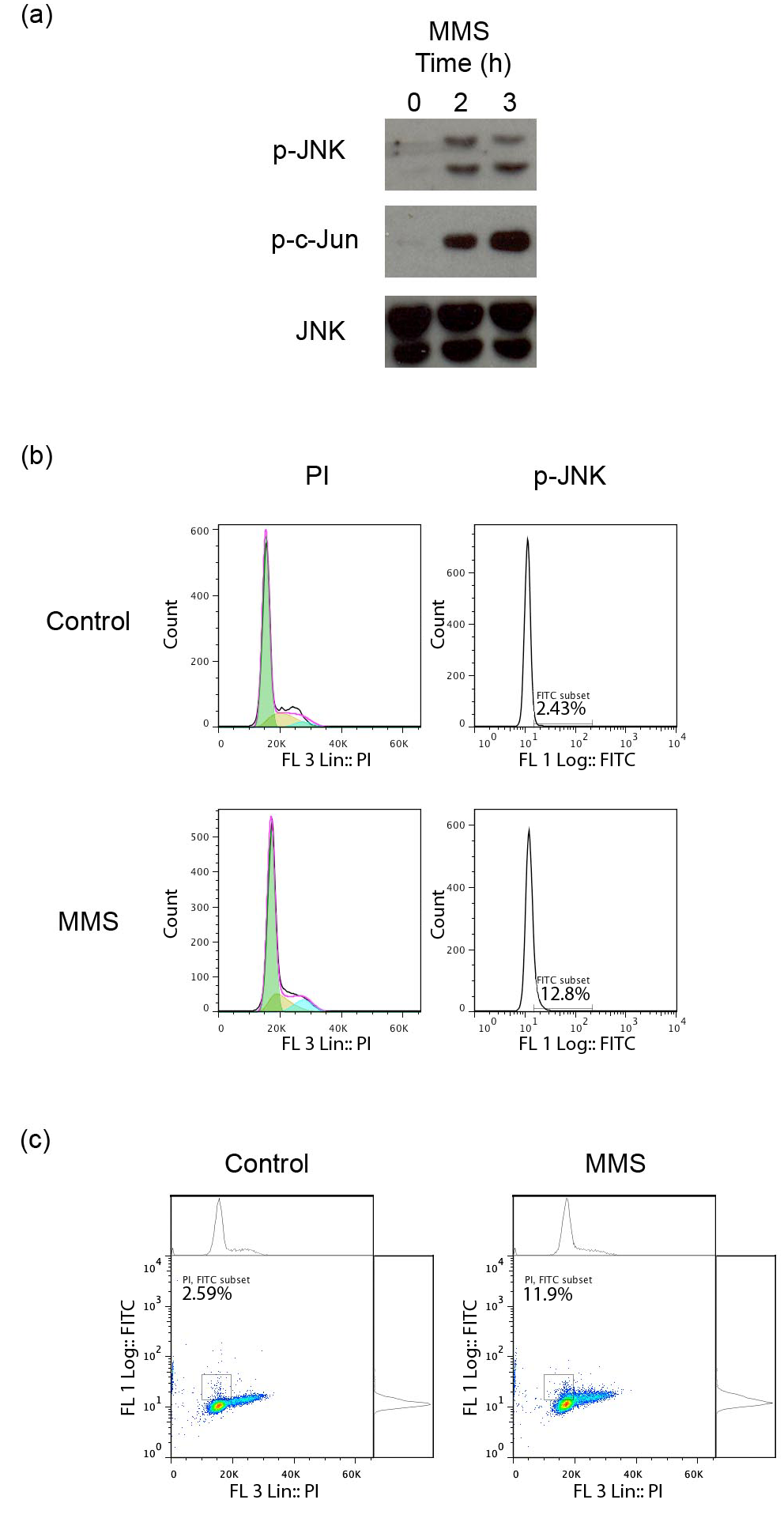

Supplement: S1 Fig — (a) Cells were treated with MMS as described and analysed by Western blotting to assess the activation of JNK using anti-p-JNK and subsequent modification of the target protein using anti-p-c-Jun. An anti-JNK antibody was used to verify equal loading. Results indicate that JNK is activated to p-JNK within 2 hours of MMS treatment, leading to c-Jun phosphorylation. (b, c) Levels of p-JNK in control and MMS treated cells were also assessed using flow cytometry. In (b) the left hand panels show propidium iodide staining of DNA to confirm that the treatment had not altered the cell cycle distribution of the population. The right hand panels show labelling of these cell populations with anti-p-JNK after control or MMS treatment. Note that the very robust Western blot signals in this well-validated pathway translate to small changes in the cytometry profiles; nonetheless, these changes are based on the measurement of labelling levels in 10,000 cells/profile and are highly significant. In (c) cells are first gated for G1 DNA content (from the propidium iodide channel on the x-axis) and then assessed for p-JNK content by counting percentage of cells above a threshold (on the y-axis) defined by a first antibody deletion control. (TIF) [file pone.0177990.s001.tif]

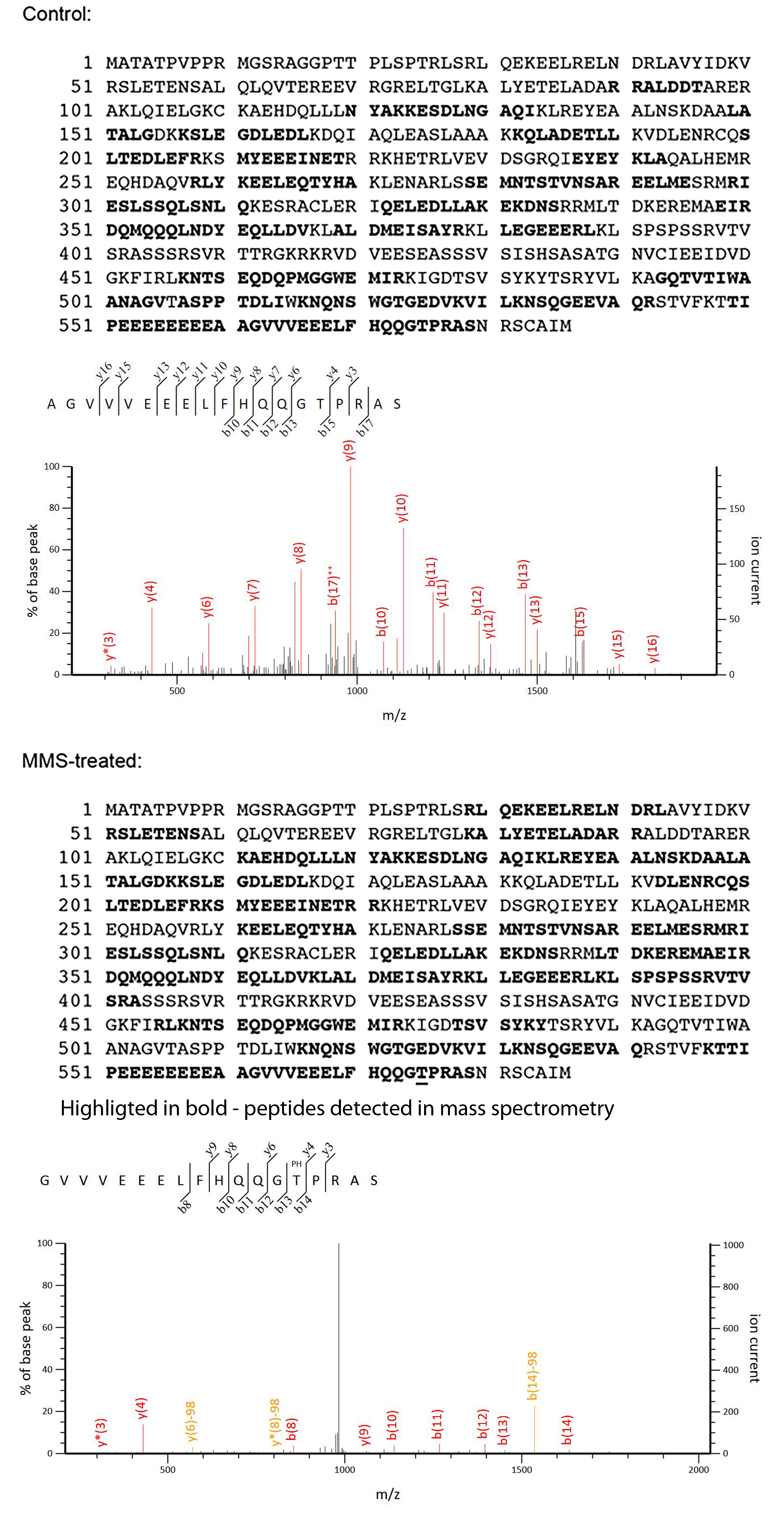

Supplement: S2 Fig — The sequence coverage of sequenced peptides (illustrated in bold) for lamin B1 is shown for the untreated control sample and the MMS treated sample. MS/MS fragmentation of lamin B1 peptide AGVVVEEELFHQQGTPRAS from the control sample shows no mass shift at threonine 575, whereas the MMS-treated sample contains the same peptide but with a phosphorylation of threonine 575. (TIF) [file pone.0177990.s002.tif]

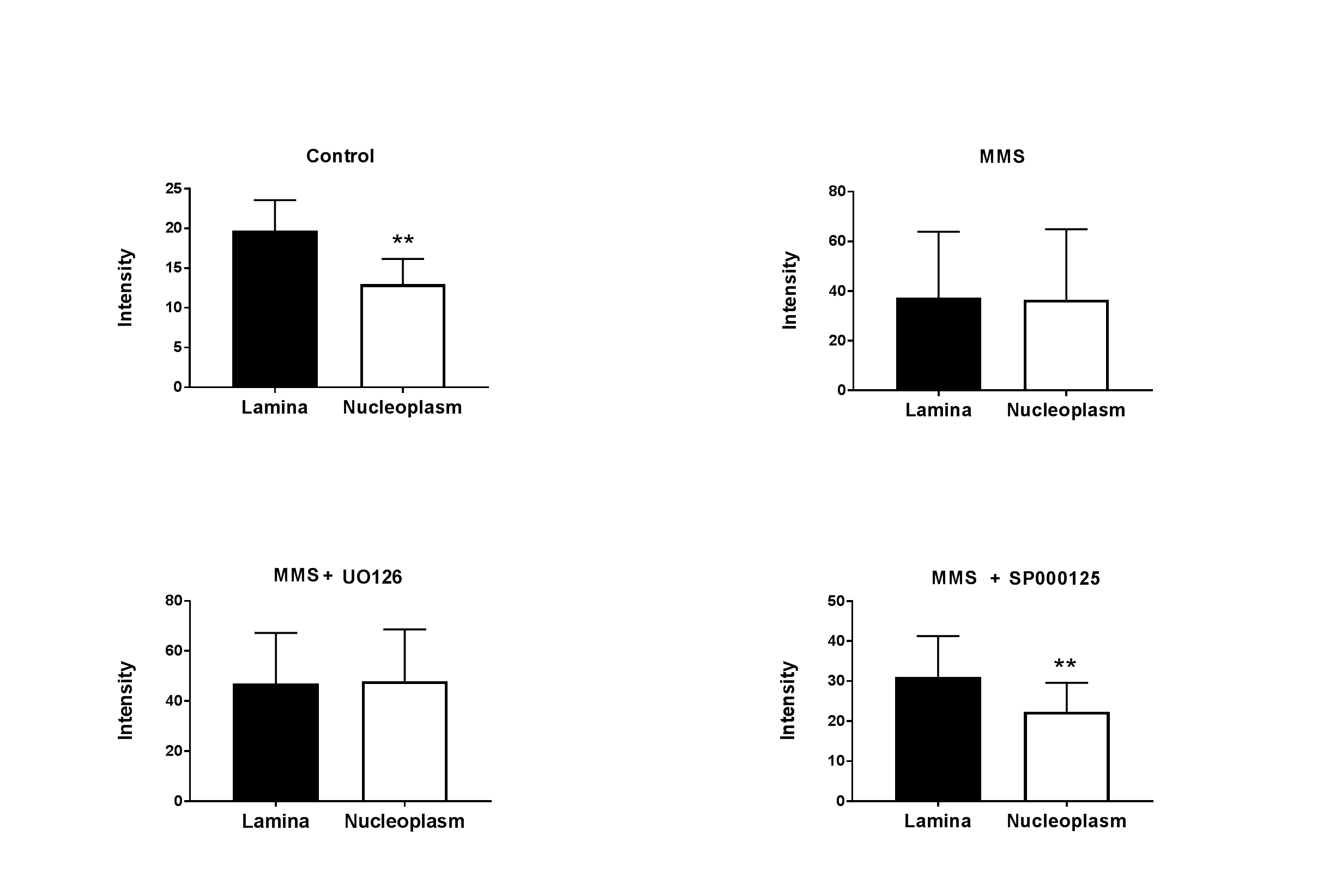

Supplement: S3 Fig — Immunofluorescence microscopy images of individual nuclei (>30 per treatment) were analysed for the distribution of Oct-1 signal intensity per unit area using or Image J. Oct-1 at the nuclear lamina area (defined by lamin B1 staining) was compared to the internal nucleoplasmic Oct1 signal by taking a ratio of the two area-normalized intensity values. Differences in Oct-1 localization at the lamina ring and in nucleoplasm are highly significant (p<0.01) in the case of control (untreated) cells (top left). However, this difference was lost after MMS treatment led to loss of peripheral Oct1 sequestration (top right). Pretreatment of the cells with ERK inhibitor UO126 did not affect this response to MMS (lower left). In contrast, pretreatment with the JNK inhibitor SP000125 abolished the release of peripheral Oct1, leaving a significant difference between peripheral and nucleoplasmic signals (lower right). This further confirms the role of JNK kinase in Oct-1 release from nuclear lamina upon MMS induced stress. (TIF) [file pone.0177990.s003.tif]

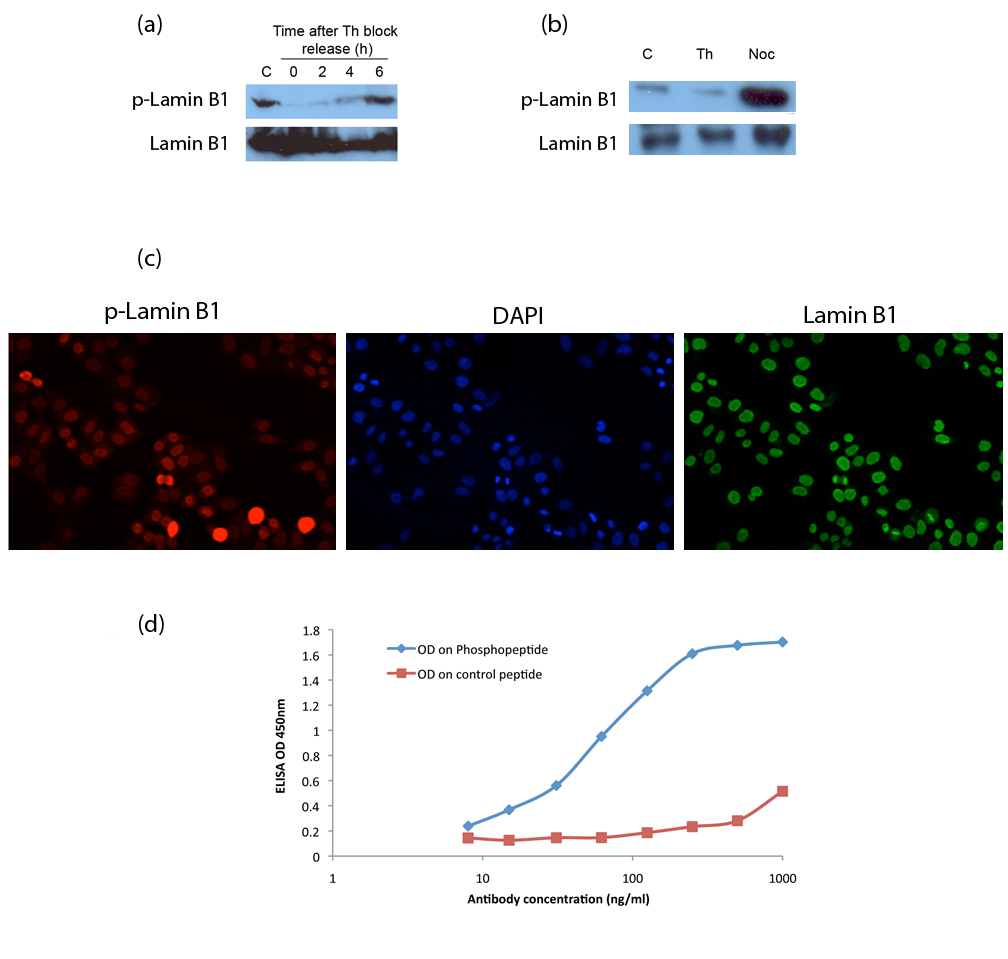

Supplement: S4 Fig — An affinity purified rabbit polyclonal anti-phospho-peptide antibody was raised against phospho-threonine 575 in lamin B1 and used to analyse phospho-T575 levels in control cells (a; lane C) and at intervals after releasing cells from a thymidine block (a) or after nocodazole treatment (b) indicating that T575 is phosphorylated during mitosis. The blot in (b) is of a construct of the 40 C-terminal amino acids of lamin B1 transfected into HeLa cells. (c) Fluorescent staining of unsynchronised HeLa cells with rabbit anti-pT575 (left panel), DAPI (centre panel) and anti- lamin B1 (right panel) showing intense phospho-T575 signals in mitotic cells. (d) ELISA confirmation of the selectivity of the affinity purified anti-phosphopeptide antibody against phospho-T575 lamin B1; control peptide (HQQGTPRASNRSC) has the same sequence as phosphopeptide (HQQG[Tp]PRASNRSC), but lacks phosphorylation at threonine T575. (TIF) [file pone.0177990.s004.tif]

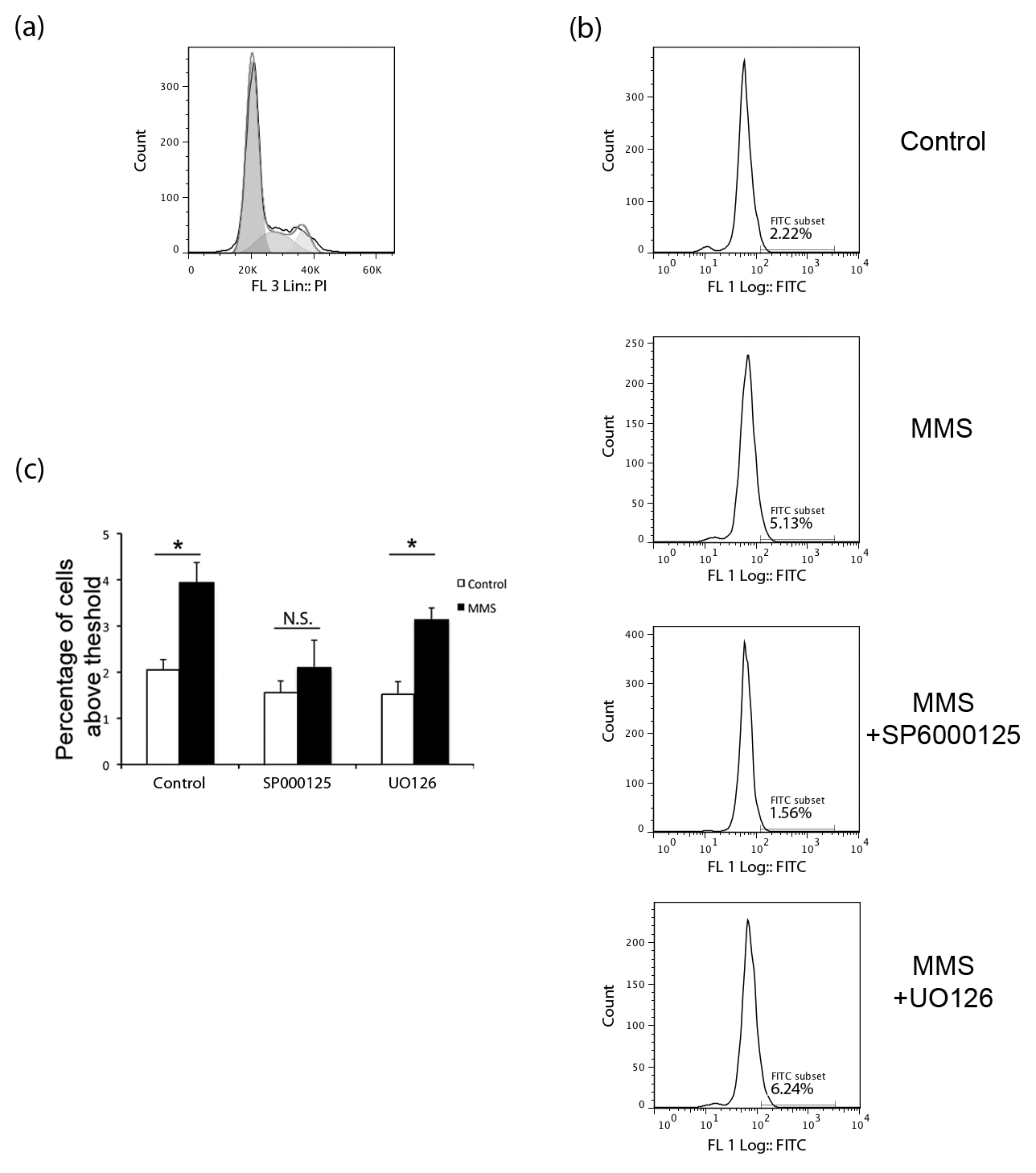

Supplement: S5 Fig — HeLa cells were incubated under the indicated conditions before being fixed and labelled with anti-phospho-lamin B1 and propidium iodide (PI) in order to analyse T575 phosphorylation and cell cycle stages respectively. (a) Cell cycle profiles based on PI staining; note the large G1 peak to the left, the G2/M peak to the right, and the intermediate saddle area of cells in S phase with intermediate DNA content. (b) G1 cells were then analysed for T575 phosphorylation levels under different treatment conditions. Data shown is a representative of at least four replicates. The threshold intensity (y-axis) for counting a cell as pT575 positive was determined as the lowest intensity value that excluded all cells in a first antibody deleted control. (There is a significant difference between control and MMS treated cells in the case of ERK inhibitor (p<0.05) and DMSO vehicle control (p<0.01) pre-treatment, but no significant difference in the case of the JNK inhibitor pre-treatment (p = 0.48). Data shown is mean +/- s.e.m. (TIF) [file pone.0177990.s005.tif]

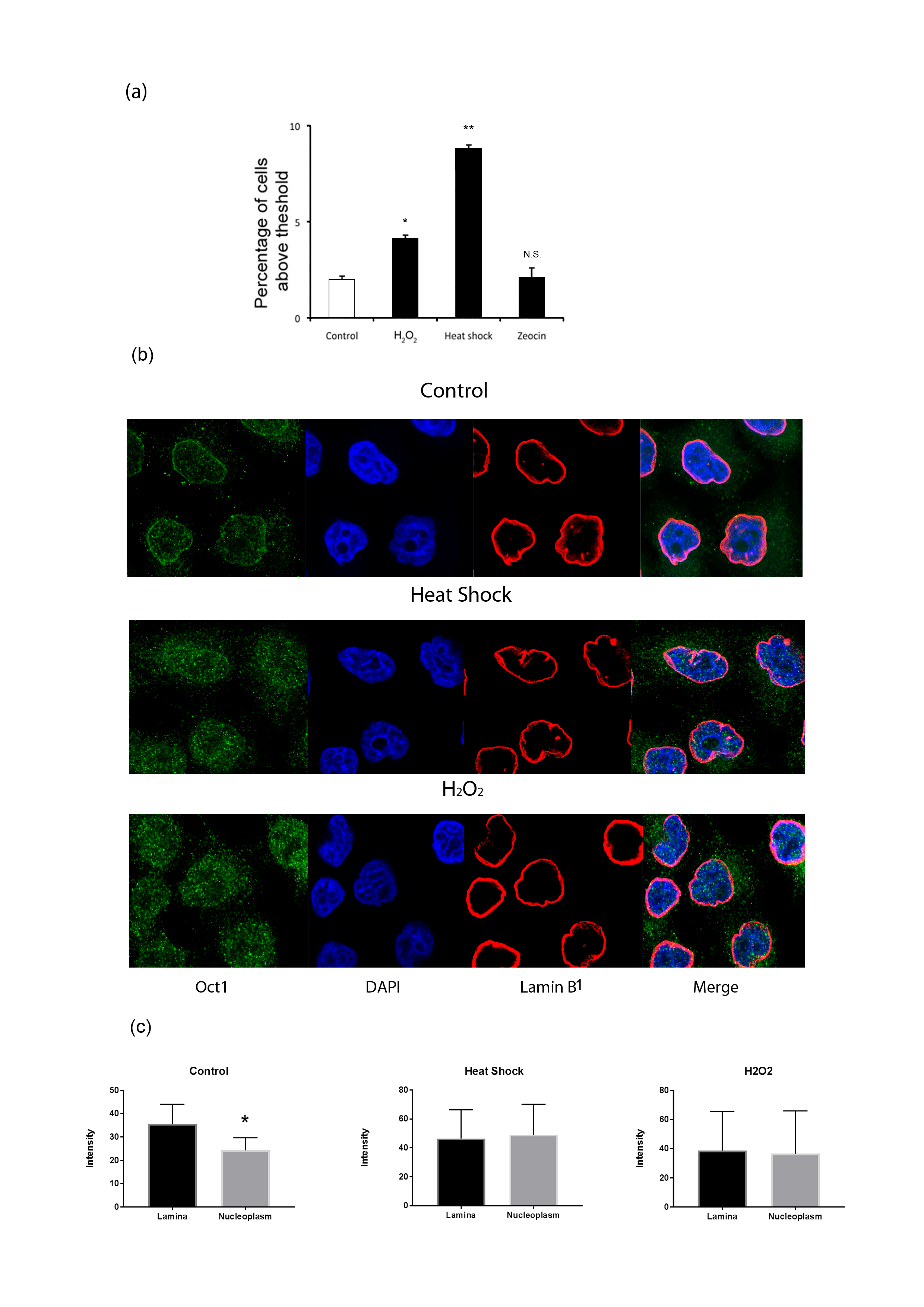

Supplement: S6 Fig — (a) Flow cytometry analysis of interphase (G1) phosphorylation events after various cell treatments. Summary of percentage of HeLa cells above T575 phosphorylation threshold (set as shown in S5 Fig and described in the legend). Heat shock and H2O2 treatments cause significant increase in interphase (G1) T575 phosphorylation, while zeocin treatments does not. (b) Microscopy evidence for the release of Oct-1 from lamin B1 after heat shock or H2O2 treatment. Co-localization of Oct-1 and lamin B1 at the NE is lost as shown by immunostaining using anti-Oct-1 and anti-lamin B1 antibodies. (c) Quantitation of experiments shown in panel (b) using Image J as described in the legend to S3 Fig. (TIF) [file pone.0177990.s006.tif]
