# Supplementary material for: Stress-induced release of Oct-1 from the nuclear envelope is mediated by JNK phosphorylation of lamin B1
Source: PLoS One. 2017 May 24;12(5):e0177990. doi: 10.1371/journal.pone.0177990 (PMC5443517; doi:10.1371/journal.pone.0177990)
Supplement: S1 Table — (DOCX) [file pone.0177990.s007.docx]

| **Name** | **Direction** | **Sequence** |
| --- | --- | --- |
| GADD45A | F | AGCTCCTGCTCTTGGAGACC |
|  | R | GCAGGATCCTTCCATTGAGA |
| ACTB | F | GCACAGAGCCTCGCCTT |
|  | R | GTTGTCGACGACGAGCG |
| GADD45A ChIP | F | CTCCTCTCAACCTGACTCCAGGAG |
|  | R | TCCGGGGTTATCCTGCCAAC |
