# Supplementary material for: Stress-induced release of Oct-1 from the nuclear envelope is mediated by JNK phosphorylation of lamin B1
Source: PLoS One. 2017 May 24;12(5):e0177990. doi: 10.1371/journal.pone.0177990 (PMC5443517; doi:10.1371/journal.pone.0177990)
Supplement: S2 Table — Data for Fig 1 (section d). (DOCX) [file pone.0177990.s008.docx]

|  | **Control**  **siRNA** | **LBsiRNA** | **LAsiRNA** | **OctsiRNA**  **LBsiRNA** |
| --- | --- | --- | --- | --- |
| GADD45A  Fold Change –Mean | 1 | 1.6521 | 1.2945 | 1.0123 |
| Standard Deviation | \|  \| \| --- \| \|  \| \| \|  \| \| --- \| \| N/A \| \|  \| \| \|  \| \|  \| | 0.3122 | 0.2809 | 0.5999 |
| Standard Error | N/A | 0.1561 | 0.1622 | 0.3463 |
