# Supplementary material for: Stress-induced release of Oct-1 from the nuclear envelope is mediated by JNK phosphorylation of lamin B1
Source: PLoS One. 2017 May 24;12(5):e0177990. doi: 10.1371/journal.pone.0177990 (PMC5443517; doi:10.1371/journal.pone.0177990)
Supplement: S3 Table — Data for Fig 1 (section e). (DOCX) [file pone.0177990.s009.docx]

|  | **Control**  **siRNA** | **LAsiRNA** | **LBsiRNA** | **OctsiRNA** | **OctsiRNA**  **LBsiRNA** |
| --- | --- | --- | --- | --- | --- |
| GADD45A Fold Change–Mean | \|  \| \| --- \| \|  \| \| 9.7886 \| \|  \| \|  \| | 12.3054 | 4.8307 | 3.0443 | 3.3188 |
| Standard Deviation | \|  \| \| --- \| \|  \| \| 1.7270 \| \|  \| \|  \| | 0.8261 | 1.7471 | 0.8588 | 0.6940 |
| Standard Error | \|  \| \| --- \| \|  \| \| 0.8635 \| \|  \| \|  \| | 0.4769 | 0.8735 | 0.4958 | 0.4007 |
