# Supplementary material for: Stress-induced release of Oct-1 from the nuclear envelope is mediated by JNK phosphorylation of lamin B1
Source: PLoS One. 2017 May 24;12(5):e0177990. doi: 10.1371/journal.pone.0177990 (PMC5443517; doi:10.1371/journal.pone.0177990)
Supplement: S4 Table — Data for Fig 2 (section c). (DOCX) [file pone.0177990.s010.docx]

|  | **MMS** | **MMS UO126** | **MMS SP6000125** |
| --- | --- | --- | --- |
| GADD45A  Fold Change –Mean | 4.0160 | 5.5300 | 1.3860 |
| Standard Deviation | \|  \| \| --- \| \| 0.7740 \|  \| \|  \| | 2.5053 | 0.2675 |
| Standard Error | 0.1473 | 0.6762 | 0.0296 |
