# Supplementary material for: Stress-induced release of Oct-1 from the nuclear envelope is mediated by JNK phosphorylation of lamin B1
Source: PLoS One. 2017 May 24;12(5):e0177990. doi: 10.1371/journal.pone.0177990 (PMC5443517; doi:10.1371/journal.pone.0177990)
Supplement: S5 Table — Data for Fig 4 (section b). (DOCX) [file pone.0177990.s011.docx]

|  | **Wild type** | **391E/393E** | **391A/393A** | **575E** | **575A** |
| --- | --- | --- | --- | --- | --- |
| GADD45A  Fold Change–Mean | 2.9761 | 3.2075 | 2.9340 | 2.3451 | 6.9339 |
| Standard Deviation | 0.7742 | 1.4881 | 0.7082 | 0.2595 | 2.9529 |
| Standard Error | 0.3160 | 0.8601 | 0.3541 | 0.1297 | 1.7068 |
