# Supplementary material for: Stress-induced release of Oct-1 from the nuclear envelope is mediated by JNK phosphorylation of lamin B1
Source: PLoS One. 2017 May 24;12(5):e0177990. doi: 10.1371/journal.pone.0177990 (PMC5443517; doi:10.1371/journal.pone.0177990)
Supplement: S6 Table — Data for Fig 2 (section d). Part a. Relative enrichment of GADD45A promoter occupancy in MMS only treated cells. Part b. Relative enrichment of GADD45A promoter occupancy in MMS plus UO126 treated cells. Part c. Relative enrichment of GADD45A promoter occupancy in MMS plus SP000125 treated cells. (DOCX) [file pone.0177990.s012.docx]

|  | **MMS** | **MMS UO126** | **MMS SP6000125** |
| --- | --- | --- | --- |
| Promoter Occupancy  Fold Change –Mean | 1.6144 | 1.8400 | 0.9305 |
| Standard Deviation | 0.1863 | 0.5550 | 0.2800 |
| Standard Error | 0.0931 | 0.2775 | 0.140 |

**Part a.**

|  | Experiment 1 | Experiment 2 | Experiment 3 | Experiment 4 |
| --- | --- | --- | --- | --- |
| Control,  ΔCт Mean | 5.646 | 6.177 | 6.402 | 6.694 |
| MMS,  ΔCт Mean | 4.835 | 5.755 | 5.649 | 5.947 |
| ΔΔCт | -0.811 | -0.422 | -0.753 | -0.747 |
| Promoter Occupancy  Fold Change | 1.7544 | 1.3397 | 1.6852 | 1.6782 |

**Part b.**

|  | Experiment 1 | Experiment 2 | Experiment 3 | Experiment 4 |
| --- | --- | --- | --- | --- |
| Control,  ΔCт Mean | 6.324 | 5.834 | 6.523 | 6.728 |
| MMS, UO126  ΔCт Mean | 5.343 | 5.599 | 5.757 | 5.401 |
| ΔΔCт | -0.981 | -0.235 | -0.766 | -1.327 |
| Promoter Occupancy  Fold Change | 1.9738 | 1.1769 | 1.7005 | 2.5088 |

**Part c.**

|  | Experiment 1 | Experiment 2 | Experiment 3 | Experiment 4 |
| --- | --- | --- | --- | --- |
| Control,  ΔCт Mean | 4.660 | 5.921 | 6.254 | 6.377 |
| MMS, SP000125  ΔCт Mean | 4.998 | 5.568 | 6.911 | 6.349 |
| ΔΔCт | 0.338 | -0.353 | 0.657 | -0.028 |
| Promoter Occupancy  Fold Change | 0.7911 | 1.2772 | 0.6341 | 1.0195 |
