# Supplementary material for: Stress-induced release of Oct-1 from the nuclear envelope is mediated by JNK phosphorylation of lamin B1
Source: PLoS One. 2017 May 24;12(5):e0177990. doi: 10.1371/journal.pone.0177990 (PMC5443517; doi:10.1371/journal.pone.0177990)
Supplement: S7 Table — (DOCX) [file pone.0177990.s013.docx]

| **Name** | **Direction** | **Sequence** |
| --- | --- | --- |
| S391A S 393A | F | GTTGAAGCTGgcaCCAgcaCCTTCTTCCCG |
| S391A S393A | R | CGGGAAGAAGGTGCTGGTGCCAGCTTCAAC |
| S391E S393E | F | GTTGAAGCTGgagCCAgagCCTTCTTCCCG |
| S391E S393E | R | CGGGAAGAAGGCTCTGGCTCCAGCTTCAAC |
| S508A | F | CTGGTGTCACAGCCgcaCCCCCAACTGACC |
| S508A | R | GGTCAGTTGGGGGTGCGGCTGTGACACCAG |
| S508E | F | CTGGTGTCACAGCCgagCCCCCAACTGACC |
| S508E | R | GGTCAGTTGGGGGCTCGGCTGTGACACCAG |
| T575A | F | CCACCAGCAGGGAgcaCCAAGAGCATCC |
| T575A | R | GGATGCTCTTGGTGCTCCCTGCTGGTGG |
| T575E | F | CCACCAGCAGGGAgagCCAAGAGCATCC |
| T575E | R | GGATGCTCTTGGCTCTCCCTGCTGGTGG |
