# Supplementary material for: Stress-induced release of Oct-1 from the nuclear envelope is mediated by JNK phosphorylation of lamin B1
Source: PLoS One. 2017 May 24;12(5):e0177990. doi: 10.1371/journal.pone.0177990 (PMC5443517; doi:10.1371/journal.pone.0177990)
Supplement: S1 Appendix — (DOCX) [file pone.0177990.s014.docx]

// this macro is written in IJ1 macro language

directory = getDirectory("Choose input files");

//Asks for the channel positions

LmnB1Channel = getNumber("In which channel is the LmnB1 staining?", 4);

Oct1Channel = getNumber("In which channel is the Oct-1 staining?", 2);

run("Set Measurements...", "area mean display redirect=None decimal=9");

list=getFileList(directory);

for (i=0;i<list.length;i++) {

open(directory+list[i]);

name = File.nameWithoutExtension();

Stack.getDimensions(width,height,channels,slices,frames);

Stack.setPosition(LmnB1Channel, 1, 1);

//thresholds the LmnB1 image based on the default algorithm and creates the ROI of the LmnB1 (ring) and the ROI of the nucleoplasm

selectWindow(name + ".tif");

run("Select All");

setAutoThreshold("Default dark");

selectWindow(name + ".tif");

run("Create Mask");

run("Analyze Particles...", "size=10.00-Infinity include");

selectWindow("mask");

run("Duplicate...", "title=mask-1");

//creates the ROI for the LmnB1

selectWindow("mask");

run("Create Selection");

roiManager("Add");

//creates the ROI for the nucleoplasm

selectWindow("mask-1");

run("Analyze Particles...", "size=10.00-Infinity include add");

roiManager("Select", 1);

run("Clear Outside");

run("Select All");

run("Invert");

run("Analyze Particles...", "size=10.00-Infinity exclude include add");

roiManager("Select", 1);

roiManager("Delete");

//displays the LmnB1 and Oct1 images

selectWindow(name + ".tif");

Stack.setPosition(LmnB1Channel, 1, 1);

run("Duplicate...", "title=[for validation]");

setLocation(500, 1, 400, 400);

roiManager("Select", 0);

selectWindow(name + ".tif");

Stack.setPosition(Oct1Channel, 1, 1);

roiManager("Select", 0);

selectWindow(name + ".tif");

setLocation(1, 1, 400, 400);

//If the user accepts the ROI then the area size and mean intensities are measured in the ROIs of Oct-1 channel

//If the ROI is rejected or the creation of the ROI fails it creates a 1x1 square in order to identify corrupted data

Boolean1 = getBoolean("Do you accept the ROI for the LmnB1 ring?");

if (Boolean1 == true) {

roiManager("Measure");

roiManager("Select", 0);

roiManager("Delete");

selectWindow("mask");

close();

m = roiManager("count");

if (m > 0) {

Boolean2 = getBoolean("Do you accept the ROI for the nucleoplasm?");

if (Boolean2 == true) {

roiManager("Measure");

roiManager("Deselect");

roiManager("Delete");

run("Close All");

} else {

roiManager("Select", 0);

roiManager("Delete");

makeRectangle(1, 1, 1, 1);

roiManager("Add");

roiManager("Select", 0);

roiManager("Measure");

roiManager("Deselect");

roiManager("Delete");

run("Close All");

}

} else {

makeRectangle(1, 1, 1, 1);

roiManager("Add");

roiManager("Select", 0);

roiManager("Measure");

roiManager("Deselect");

roiManager("Delete");

run("Close All");

}

} else {

roiManager("Deselect");

roiManager("Delete");

run("Close All");

}

}
